# Wnt3-mediated fibrosis and carcinogenesis of lung squamous cell carcinoma in idiopathic pulmonary fibrosis

**DOI:** 10.1016/j.isci.2026.115667

**Published:** 2026-04-09

**Authors:** Atsushi Matsuoka, Kazuhiko Shien, Shuta Tomida, Masayoshi Ohki, Hidejiro Torigoe, Kazuya Hisamatsu, Ryota Fujiwara, Kosei Ishimura, Shunsuke Mori, Ryunosuke Fujii, Asuka Mimata, Kazuhiro Okada, Ryo Yoshichika, Mao Yoshikawa, Yuma Fukumoto, Haruchika Yamamoto, Kumi Nakajima, Shin Tanaka, Ken Suzawa, Kentaroh Miyoshi, Mikio Okazaki, Seiichiro Sugimoto, Hirofumi Inoue, Daisuke Ennishi, Shinichi Toyooka

**Affiliations:** 1 Department of General Thoracic Surgery and Breast and Endocrinological Surgery, Okayama University Graduate School of Medicine, Dentistry and Pharmaceutical Sciences, Okayama, Japan; 2 Center for Comprehensive Genomic Medicine, Okayama University Hospital, Okayama, Japan

**Keywords:** Oncology, Respiratory medicine, Pathology, Precision medicine, Target identification, Systems biology, Cancer, Omics, Transcriptomics

## Abstract

Idiopathic pulmonary fibrosis (IPF) increases the risk of lung squamous cell carcinoma (LUSC), yet its molecular pathogenesis remains unclear. We conducted multi-omics analysis, including single-cell RNA sequencing and digital spatial profiling, on LUSC specimens from seven patients with usual interstitial pneumonia (UIP). In UIP lung tissue, metaplastic basal cells arising from the transdifferentiation of alveolar type 2 (AT2) cells were increased. LUSC tumors arising within UIP exhibited molecular profiles and trajectory dynamics suggesting derivation from these metaplastic basal cells. Both UIP-affected tissue and associated tumors showed activation of Wnt signaling, particularly *WNT3* expression. Additionally, enrichment of the nuclear factor erythroid 2-related factor 2 (NRF2)-linked antioxidant response was observed in LUSC within UIP. Targeting Wnt/β-catenin signaling restored the sensitivity of these stress-adapted cancer cell lines to oxidative damage. These findings suggest that LUSC within UIP originates from AT2-derived metaplastic basal cells and involves aberrant Wnt3 activation, linking fibrosis to carcinogenesis and highlighting a potential therapeutic strategy.

## Highlights

• LUSC in UIP lesions originates from AT2-derived metaplastic basal cells
• A Wnt-upregulated antioxidant tumor subclone selectively expands in the UIP fibrotic niche
• Shared *WNT3* upregulation links IPF pathogenesis to LUSC carcinogenesis
• Targeting Wnt pathway restores oxidative stress sensitivity in IPF-associated LUSC

## Graphical abstract

**Figure:**
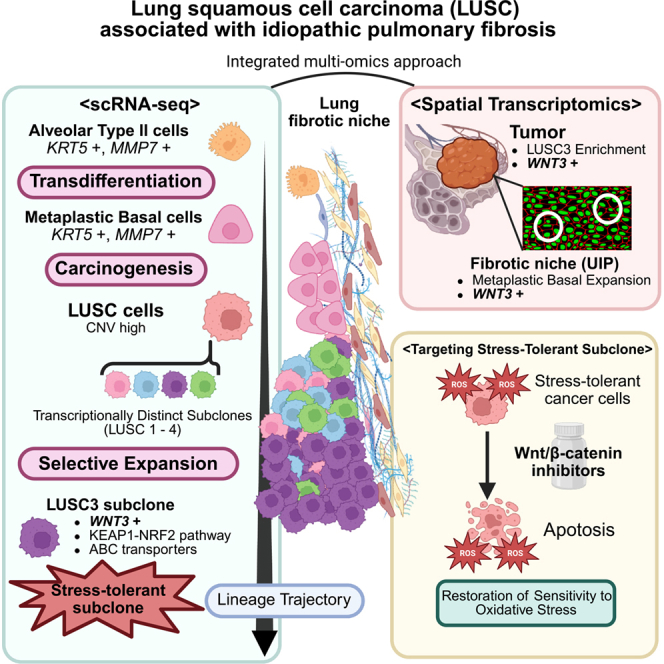

## Introduction

Patients with interstitial lung disease (ILD), especially idiopathic pulmonary fibrosis (IPF), have a significantly increased risk of developing lung cancer.^1^^,^^2^^,^^3^ A meta-analysis reported that 13.5% of patients with IPF developed non-small cell lung cancer (NSCLC).^4^ Among these, lung squamous cell carcinoma (LUSC) was the most frequent histologic variant (37.8%), followed by lung adenocarcinoma (LUAD), at 30.8%.^4^ The presence of lung cancer in IPF patients is associated with a poor prognosis, characterized by higher lung cancer-specific mortality and shorter overall survival, even in early-stage NSCLC.^5^^,^^6^ Most lung cancer patients with IPF have reduced pulmonary function and poor performance status, making standard lung cancer treatments difficult. Furthermore, life-threatening acute exacerbations of IPF can be triggered by cancer treatments including radiotherapy, conventional chemotherapy, tyrosine kinase inhibitors, immune checkpoint inhibitors, and surgery, thus significantly limiting therapeutic options. Therefore, there is a pressing need for innovative therapeutic strategies to improve the prognosis of patients with coexisting lung cancer and IPF.

Shared risk factors such as smoking, environmental exposure, aging, and genetic predispositions are believed to contribute to both IPF and lung cancer.^7^ A comprehensive review has recently updated the epidemiological and pathobiological links between these two conditions, highlighting shared cellular, molecular, and epigenetic alterations.^8^ Furthermore, recent Mendelian randomization studies have elucidated causal associations between systemic inflammatory regulators^9^ or specific immune cell immunophenotypes^10^ and the risk of IPF. These findings suggest that the dysregulated inflammatory microenvironment associated with IPF pathogenesis may underpin the development of concomitant lung cancer. Likewise, several overlapping pathogenic mechanisms have been proposed, including epigenetic and genetic alterations, dysregulated microRNA (miRNA) expression, and cellular or molecular aberrations such as impaired regulatory signaling, delayed apoptosis, disrupted cell-to-cell communication, and activation of specific signaling pathways.^11^^,^^12^ NSCLC in patients with IPF often exhibits a distinct molecular profile, including a lower frequency of *EGFR* mutations and a higher incidence of *KRAS* G12C and *BRAF* mutations, compared to NSCLC in patients without IPF.^13^^,^^14^

The pathogenesis of IPF is complex, involving responses to epithelial injury and a cascade of molecular mechanisms leading to fibrosis.^7^ Histologically, the usual interstitial pneumonia (UIP) pattern—characterized by dense fibrosis, bronchiolectasis, and honeycombing—is the most common site for lung cancer development in IPF patients. Lesions tend to occur more frequently in the lower lobes than in the upper lobes.^15^^,^^16^ These findings suggest that the mechanisms of carcinogenesis may differ between tumors arising within UIP regions and those occurring outside them.

In this study, we performed multi-omics analyses comparing both tumor and non-tumor lung tissues from LUSC arising within and outside UIP regions. We established robust cell signatures derived from distinct cell populations validated by carcinogenesis and differentiation trajectories and then rigorously mapped these populations to the fibrotic niche, using spatial transcriptomics via a deconvolution workflow. By comparing their molecular landscapes with this high-resolution approach, we identified key factors contributing to the carcinogenic origin of LUSC that develops in UIP-affected areas. These findings have important implications for both the prevention of carcinogenesis in IPF patients and the development of novel therapeutic strategies for lung cancer in the context of IPF.

## Results

### Patient cohort

We retrospectively selected seven patients with primary LUSC exhibiting a UIP pattern on preoperative chest CT (Table S1). All were men, aged 56–79 years, with a history of smoking (36–82.5 pack-years). The pathological stage ranged from IA2 to IIIB.

### Single-cell transcriptomic profiling reveals aberrant airway-like epithelial expansion in UIP lesions

**Figure 1:**
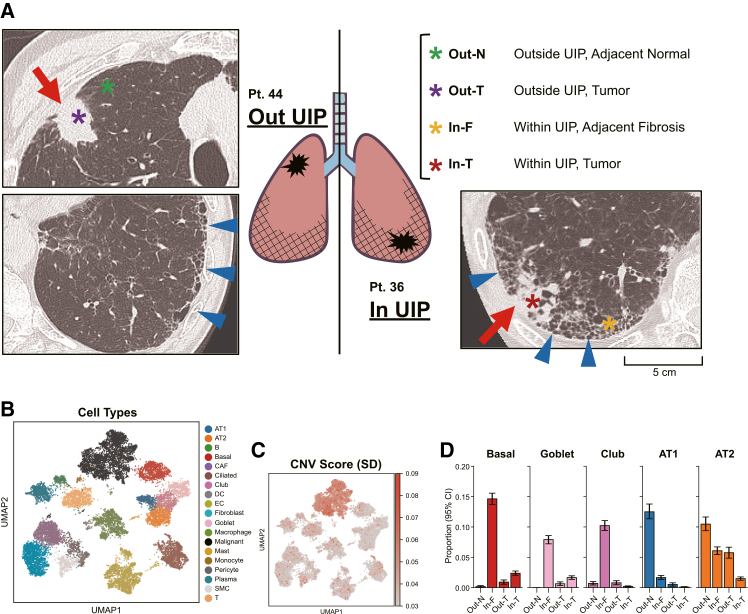
Single-cell transcriptomic profiling of LUSC tumors within and outside UIP regions (A) Preoperative chest CT images and schematic showing sample collection sites for scRNA-seq from two LUSC patients: one with tumor within a UIP lesion (In UIP), and the other with tumor outside UIP (Out UIP). Tumor regions (In-T and Out-T) and adjacent non-tumor tissues (fibrotic [In-F] and normal [Out-N]) were collected from frozen lung tissue for analysis. Colored asterisks indicate sampling sites, and blue arrowheads highlight radiological features of UIP. Scale bars represent 5 cm. (B) UMAP plot showing cell type annotations from all four samples. AT1, alveolar type 1 cell; AT2, alveolar type 2 cell; B, B cell; Basal, basal cell; CAF, cancer-associated fibroblast; Ciliated, ciliated epithelial cell; Club, club cell; DC, dendritic cell; EC, endothelial cell; Goblet, goblet cell; Malignant, malignant tumor cell; Mast, mast cell; Plasma, plasma cell; SMC, smooth muscle cell; T, T cell. (C) UMAP plot displaying the standard deviation (SD) of inferred CNVs for each cell. Cells from Out-N regions were used as the normal reference. High CNV scores highlight malignant cell populations. (D) Bar plots displaying the proportions of five epithelial cell types in each sample. Error bars represent 95% confidence intervals estimated via bootstrap resampling (*n* = 1,000). The In-F sample, despite being derived from peripheral lung tissue, exhibited a markedly increased proportion of airway-like epithelial cells (Basal, Goblet, and Club), with a reduction in alveolar epithelial cells (AT1 and AT2). LUSC, lung squamous cell carcinoma; UIP, usual interstitial pneumonia; UMAP, uniform manifold approximation and projection; CNVs, copy number variations.

To determine the composition and gene expression profiles of tumors arising within versus outside UIP regions at single-cell resolution, we performed single-cell RNA sequencing (scRNA-seq) on fresh-frozen tissues from two representative patients. From each, we prepared tumor (In-T [tumor] and Out-T) and adjacent non-tumor regions (In-F [fibrosis] and Out-N [normal]) (Figure 1A). After rigorous quality control and integration with Scanpy,^17^ we retained 9,262 cells from In-T, 6,293 from In-F, 2,774 from Out-T, and 3,030 from Out-N regions. Uniform manifold approximation and projection (UMAP) delineated 17 distinct cell populations—including epithelial cells, mesenchymal cells, immune cells, and malignant cells—annotated based on established marker genes and cross-validated with automated CellTypist^18^ predictions (Figures 1B, S1A, and S1B). To discriminate malignant cells, we inferred copy number variation (CNV) profiles, using inferCNV. Mapping the standard deviation (SD) of inferred CNVs onto the UMAP revealed high CNV scores localized to putative malignant clusters (Figure 1C).

Then, we quantified the principal epithelial lineages (alveolar type 1 [AT1], alveolar type 2 [AT2], basal, club, and goblet cells) in each sample. Compared with Out-N, the In-F region exhibited a marked expansion of basal, club, and goblet cells, concurrent with a reduction in AT1/AT2 cells (Figure 1D). This shift may suggest aberrant airway-like epithelial differentiation in fibrotic lung parenchyma.^19^^,^^20^^,^^21^ The overall distribution of all annotated cell types across the four samples is summarized in Figure S1C.

### Identification of four malignant subclones with distinct transcriptomic phenotype

**Figure 2:**
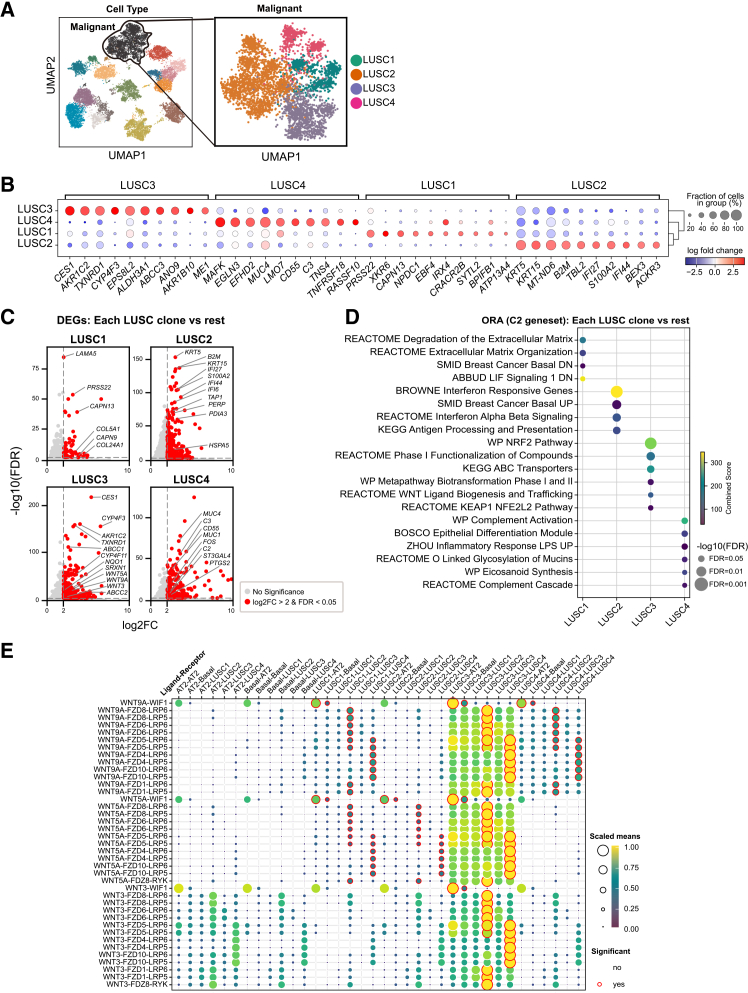
Identification and characterization of transcriptionally distinct malignant subclones (A) Subclustering of malignant cells from two tumor samples reveals four discrete subpopulations (LUSC1–LUSC4). (B) Dot plot of log fold change (FC) for the top 10 DEGs in each subclone versus all others. Dot size reflects the fraction of cells expressing the gene; color indicates logFC. Hierarchical clustering of subclones is shown to the right. (C) Volcano plots for each subclone versus the remainder, with DEGs highlighted in red. Key marker genes that may help distinguish each subclone are annotated. (D) Over-representation analysis (ORA) of C2-curated gene sets for each subclone. Dots represent significantly enriched pathways (FDR < 0.05); size denotes significance, and color indicates combined score. (E) Dot plot of Wnt ligand-receptor interactions among LUSC1–LUSC4, basal, and AT2 cells. Dot size and color reflect the scaled mean expression of ligand-receptor pairs; red outlines mark statistically significant interactions (*p* < 0.05). LUSC3 shows elevated Wnt ligand expression and strong autocrine and paracrine signaling with neighboring epithelial populations.

To dissect transcriptomic diversity within tumors, we subclustered all malignant cells from the two scRNA-seq samples, from which we inferred four transcriptionally distinct subclones (LUSC1–4) (Figure 2A). Differential expression analysis identified the top ten marker genes for each subclone compared to the rest, and their largely non-overlapping expression patterns across cells confirmed that each subclone harbored a discrete transcriptional program rather than artifactual over-clustering (Figure 2B). Volcano plots show differentially expressed genes (DEGs) (log_2_ fold change [FC] > 2 and false discovery rate [FDR] < 0.05) of each subclone (Figure 2C), and over representation analysis (ORA) of each set of DEGs (Figure 2D) delineated each phenotype.

LUSC1 showed upregulated extracellular matrix (ECM) structural genes (e.g., *LAMA5*, *COL5A1*, and *COL24A1*)^22^ and proteases (e.g., *PRSS22*, *CAPN9*, and *CAPN13*)^23^^,^^24^ while exhibiting downregulation of basal-differentiation and LIF signaling, defining an invasive, ECM remodeling phenotype. LUSC2 exhibited co-expression of basal keratins and adhesion factors (e.g., *KRT5*, *KRT15*, and *PERP*) with interferon-stimulated genes (ISGs) (e.g., *IFI6*, *IFI27*, and *IFI44*) and antigen-presentation genes (e.g., *TAP1*, *B2M*, *PDIA3*, and *HSPA5*),^25^ along with *S100A2*, a marker of well-differentiated squamous cell carcinoma,^26^ characterizing a “classical” and keratinized subclone that simultaneously engages innate immune programs. LUSC3 showed uniquely enriched phase 1 detoxification enzymes (e.g., *CES1*, *AKR1C2*, *CYP4F3*, and *CYP4F11*),^27^^,^^28^^,^^29^ KEAP1-NRF2 antioxidant targets (e.g., *TXNRD1*, *SRXN1*, and *NQO1*),^30^ phase 2 detoxification enzymes,^31^ ABC transporters (e.g., *ABCC1* and *ABCC2*) upregulated by oxidative stress,^32^ and Wnt ligands (e.g., *WNT3*, *WNT5A*, and *WNT9A*), signifying a coordinated antioxidant/detoxification and Wnt-enriched program. LUSC4 exhibited upregulated complement components (e.g., *C3* and *C2*), along with the inhibitory regulator *CD55*, indicating activation of the complement system with protective feedback that may contribute to immune evasion. CD55 is known to protect cancer cells from complement attack and has been implicated in immune escape across various tumor types.^33^ Genes associated with inflammatory signaling (e.g., *PTGS2* and *FOS*)^34^ were also elevated in LUSC4, consistent with innate immune activation and the eicosanoid biosynthetic pathway. In parallel, it retained epithelial differentiation with high expressions of mucins (e.g., *MUC1* and *MUC4*) and mucin-modifying enzymes such as ST3GAL4,^35^ characterizing a well-differentiated, immune-reactive subclone consistent with an early stage of tumor development.

To delineate intercellular signaling, we applied CellPhoneDB^36^ to infer ligand-receptor interactions across all epithelial populations. As shown in Figure 2E, we specifically extracted interactions involving *WNT3*, *WNT5A*, and *WNT9A*, the ligands highly upregulated in LUSC3, highlighting robust autocrine signaling in this subclone. Figure S2A expands on this by showing the full complement of Wnt family ligand-receptor pairs that reached statistical significance (*p* < 0.05) in at least one pairwise comparison among the LUSC1–4 subclones, basal, and AT2 cells. Together, these analyses revealed that LUSC3 not only drives its own Wnt-Frizzled axis but also engages in paracrine Wnt signaling with neighboring epithelial populations—an integrated signaling niche that likely cooperates with its KEAP1-NRF2-mediated antioxidant and detoxification program to bolster oxidative stress resilience in the fibrotic tumor microenvironment.

### Prediction of cellular dynamics reveals late-phase dominance of an antioxidant/detoxification/Wnt-enriched subclone in tumor within UIP

**Figure 3:**
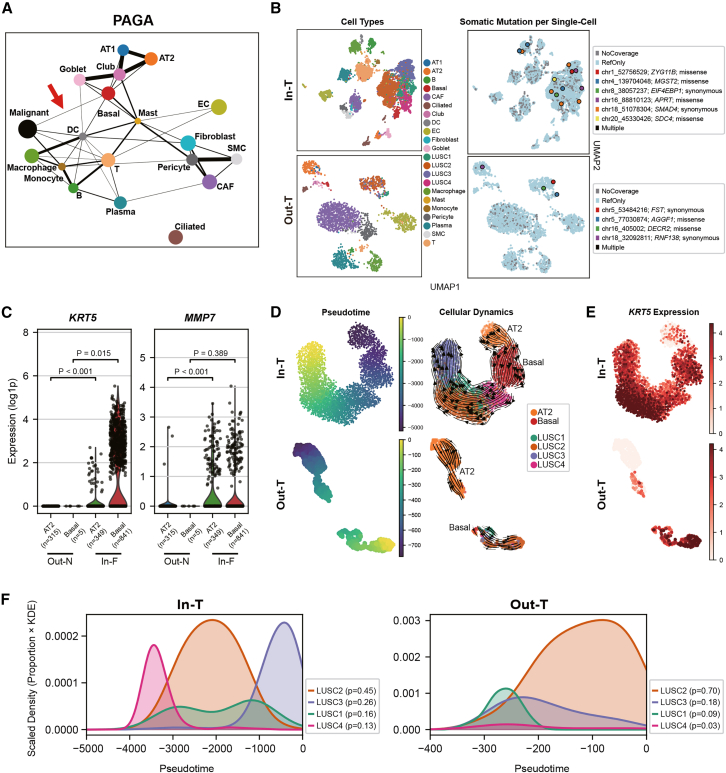
PAGA, cellular phenotype, and cellular trajectory analysis identify AT2-derived metaplastic basal cells as the origin and LUSC3 dominance in tumor within UIP (A) PAGA shows connectivity among cell types. Each node represents a cell population, and edge thickness reflects the confidence of connection. When the edge threshold was incrementally increased to infer the potential origin of malignant cells, only the connection between malignant and basal cells remained among all non-malignant epithelial types (Basal, Club, Goblet, AT1, and AT2). (B) UMAP plot of cell type annotations (left) and distribution of somatic mutations from paired WES (right). Cells with no coverage at any variant site are shown in gray, and cells with only reference reads are shown in light blue. Cells set to be labeled as “multiple” (harboring ≥2 detected mutations) were not observed. (C) Violin plots showing the expression of *KRT5* and *MMP7* in basal and AT2 cells from non-tumor tissues. Basal cells in the In-F region showed significantly higher *MMP7* expression than the rare basal cells in the Out-N, indicating a pre-existing metaplastic phenotype in the UIP background. Statistical significance was determined by the Wilcoxon rank-sum test; exact *p* values and the number of cells (*n*) are indicated. (D) Pseudotime and vector-field dynamics inferred using scTour. Left: pseudotime values projected onto the UMAP for In-T and Out-T regions. Right: smooth and continuous streamlines and arrows depict transitions. (E) Feature plots showing *KRT5* expression dynamics along the inferred trajectory. (F) KDEs of pseudotime for LUSC subclones in each tumor, scaled by subclone abundance. Early pseudotime values overlapped for all subclones, LUSC2 peaked at intermediate stages, and only In-T showed a pronounced late-phase peak of LUSC3, indicating its dominant emergence in tumor within UIP. PAGA, partition-based graph abstraction; UMAP, uniform manifold approximation and projection; WES, whole-exome sequencing; KDEs, Kernel density estimates.

To uncover the cellular origin of malignant cells, we constructed a partition-based graph abstraction (PAGA) across all annotated cell types. The PAGA demonstrated strong connectivity between malignant cells and basal cells, with only weak or absent edges to other epithelial populations. By increasing the edge confidence threshold, all low confidence links were removed except the basal-malignant edge, implicating basal cells as the most likely cell of origin for the malignant population (Figure 3A). This result aligns with reports that human basal stem cells harbor squamous cell carcinoma transcriptional signatures due to error-prone DNA repair,^37^ with genetically engineered mouse models showing that SOX2-driven squamous cell carcinoma arises from basal cells.^38^ We next projected somatic variants from paired tumor-normal whole-exome sequencing (WES) onto the individual single-cell UMAP. Cells with private tumor mutations were localized almost exclusively to the malignant cluster (Figure 3B). Because these mutations were uniformly distributed across all subclones, we could not resolve intratumoral heterogeneity at the level of WES-detected variants. To further characterize the specific nature of these basal cells, we focused on the profile of basal and AT2 cells. It has been reported that in response to severe alveolar injury including UIP pattern fibrosis, “metaplastic basal cells” originate from the transdifferentiation of AT2 cells and are characterized by the *de novo* expression of metaplastic marker genes such as *KRT5* and *MMP7*.^21^ In the fibrotic background (In-F), AT2 cells exhibited a statistically significant upregulation of these specific metaplastic markers compared to normal AT2 cells (Out-N) (Figure 3C). This suggests an active transitional state from alveolar to basal lineage within the fibrotic niche. Consistent with this transition, basal cells in the In-F region also displayed a marked upregulation of these markers. In contrast, the normal alveolar background (Out-N) contained an extremely low number of basal cells, which lacked this metaplastic signature. These findings suggest that the expansion of basal-like cells observed in the UIP lesions represents a metaplastic transition from AT2. However, this distinct expression pattern was not conserved in the tumor tissues (Figure S3A), likely reflecting the confounding effects of the tumor microenvironment.

To address the dynamic transition from these origins, we used scTour^39^ to infer pseudotime trajectories and vector-field dynamics, in both In-T and Out-T regions. In Figure 3D, these pseudotime values and streamlines are projected onto the UMAP embedding for each region. Notably, the In-T trajectory revealed a continuous gradient originating from AT2, transitioning through a basal-like state consistent with this metaplastic conversion and extending into the malignant subclones. In contrast, the Out-T trajectory showed a disconnection between AT2 and the basal/tumor lineage, consistent with an origin from resident airway basal cells. This contrast was further supported by projecting the expression of the metaplastic marker *KRT5* onto the trajectory (Figure 3E). In the In-T region, *KRT5* expression increased continuously from the AT2 cluster toward the basal lineage, visually confirming the progressive metaplastic transdifferentiation. Conversely, in the Out-T region, although a minor sub-cluster within AT2 cluster exhibited mild *KRT5* expression—likely reflecting reactive alveolar plasticity within the tumor microenvironment—there was no clear continuity in expression connecting them to the basal or tumor cells, supporting lineage disconnection. These results strongly support the hypothesis that AT2-derived metaplastic basal cells, which pre-exist in the fibrotic niche, serve as the cellular origin of LUSC within UIP. In the density plots of pseudotime distributions (Figure 3F), the earliest values in both In-T and Out-T samples show overlapping curves for all subclones, indicating their co-emergence at tumor onset. At intermediate pseudotime values, LUSC2, the “classical” squamous subclone, predominates in both regions, reflecting the typical evolution of LUSC. This pattern parallels an *in vivo* multi-color lineage tracing study showing that early squamous cell carcinoma arises via the polyclonal expansion of multiple clones before one ultimately dominates.^40^ In contrast, only the In-T sample displayed a pronounced late-phase peak of LUSC3. This selective enrichment of LUSC3, which is characterized by its KEAP1-NRF2-mediated antioxidant and detoxification program alongside elevated Wnt ligand production, suggests a specialized adaptation to the oxidative stress conditions unique to the UIP-associated fibrotic microenvironment.

### Deconvolution of spatial transcriptomics confirms aberrant metaplastic epithelial expansion and LUSC3 subclone enrichment in UIP-associated regions

**Figure 4:**
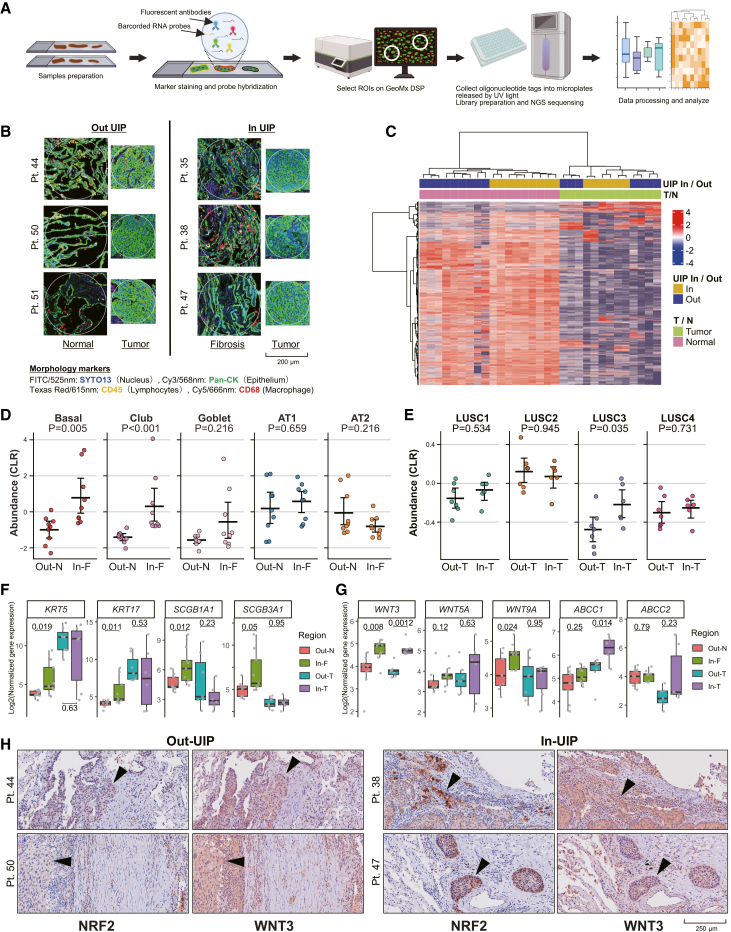
Spatial transcriptomics confirm aberrant metaplastic epithelial expansion and LUSC3 subclone enrichment in UIP-associated regions (A) Schematic workflow of digital spatial transcriptomics, using the GeoMx Digital Spatial Profiler (DSP). (B) Definition of regions of interest (ROIs) on GeoMx DSP. ROIs were defined to include core tumor regions (center of tumor nests, Pan-CK highly positive with nuclear enlargement), adjacent fibrotic areas (In-F; from three In-UIP patients) (relatively Pan-CK negative, SYTO13 dense), and morphologically normal alveolar regions (Out-N; from three Out-UIP patients). Scale bars represent 200 μm. (C) Unsupervised hierarchical clustering heatmap of the top 25% high-coefficient of variation (CV) genes across tumor core and adjacent non-tumor ROIs. Gene expression values are scaled by row. Non-tumor ROIs separate clearly by UIP status, reflecting distinct transcriptomic profiles. (D) Cell type relative abundance in adjacent non-tumor ROIs. Point-range plots display the centered-log-ratio (CLR)-transformed expected counts. Individual dots represent each ROI. Horizontal bars represent the mean, and error bars indicate the 95% bootstrapped confidence interval (CI) calculated from 1,000 iterations. The resulting CLR values were used for statistical tests (Wilcoxon rank-sum test) between two groups. Basal and club cells are enriched in In-F. (E) Cell type relative abundance in core tumor ROIs. Only the LUSC3 shows significant enrichment in In-T. Plotting conventions and statistical tests are the same as in (D). (F) Expression of specific markers across regions. Boxplots depict normalized gene expression of metaplastic basal cell markers (*KRT5* and *KRT17*) and club cell markers (*SCGB1A1* and *SCGB3A1*) in four classes. Individual ROIs are shown as gray dots. Pairwise comparisons (Out-N vs. In-F; Out-T vs. In-T) were performed by Wilcoxon rank-sum test, with *p* values displayed above each comparison. Boxes denote the median and inter-quartile range; whiskers extend to 1.5× IQR. (G) Expression of LUSC3-specific Wnt ligands (*WNT3*, *WNT5A*, and *WNT9A*) and ABC transporters (*ABCC1* and *ABCC2*) across regions. Plotting conventions and statistical tests are the same as in (F). (H) Representative immunohistochemistry of NRF2 and WNT3. The images show the tumor boundaries in patients with (In-UIP) and without (Out-UIP) fibrosis. Arrowheads indicate tumor cells at the periphery. In In-UIP cases, tumor cells at the boundary co-express WNT3 and NRF2, whereas in Out-UIP cases, NRF2 expression is largely absent in boundary regions. Scale bars represent 250 μm.

To validate and extend our single-cell observations from two cases, we applied spatial transcriptomics by using GeoMx Digital Spatial Profiler (DSP, NanoString Technologies) to a larger cohort of six LUSC patients (three within and three outside, one was overlapped to scRNA-seq). GeoMx whole-transcriptome atlas (WTA; NanoString Technologies) enables comprehensive quantification of protein-coding transcripts in spatially selected regions of tissue sections by combining barcoded probes with ultraviolet (UV) light-based oligo release (Figure 4A). Regions of interest (ROIs) were defined based on immunofluorescence staining images. Core tumor regions were manually selected at the center of tumor nests where Pan-CK expression was high and nuclei were enlarged (In-T and Out-T). Adjacent fibrotic areas came from three within-UIP patients (In-F), and morphologically normal alveolar regions were sampled from three outside-UIP patients (Out-N) (Figure 4B).

Unsupervised hierarchical clustering separated In-F from Out-N regions in non-tumor regions, reflecting the morphological and histological distinctions between within and outside UIP in comprehensive RNA expression profiles (Figure 4C). In contrast, clustering of tumor ROIs failed to clearly distinguish UIP In/Out cases. A 3D UMAP using all genes revealed only a modest tendency for tumor ROIs to segregate by UIP status, without clear separation (Figure S4A). This suggests that bulk RNA sequencing or “local-bulk” spatial transcriptomics alone cannot resolve the divergent subclone evolution and intratumoral heterogeneity we observed by scRNA-seq in In-T versus Out-T samples.

To address this, we applied Cell2location^41^ to derive reference expression signatures for each scRNA-seq-defined cell type—including the malignant subclones—and used these signatures to deconvolute the spatial transcriptomics data, thereby estimating the relative abundance of each population in every ROI. Deconvolution accuracy was validated by comparing the observed and reconstructed gene counts, confirming that the inferred cell abundances reliably reflect the spatial expression landscape (Figure S4B). In non-tumor regions, centered-log-ratio (CLR)-transformed counts revealed a significant increase in the basal and club cells in fibrotic lung (In-F) compared with outside-UIP alveoli (Out-N) (Figure 4D). These spatial abundance changes recapitulate our single-cell observations in Figure 1D, where In-F region exhibited marked expansion of metaplastic basal and aberrant secretory cell populations. Although AT2 cells showed a decreasing trend in In-F, the difference did not reach statistical significance, and AT1 cells did not show a consistent reduction in spatial abundance. This discrepancy may reflect inter-patient variability or the limitations of deconvolution in distinguishing terminally differentiated AT1 cells from aberrant intermediate populations that accumulate during impaired alveolar regeneration.^20^^,^^42^^,^^43^ In tumor cores, LUSC3 showed a significant increase in In-T versus Out-T, while other subclones exhibited no significant regional differences (Figure 4E). This finding is consistent with the late-phase dominance of LUSC3 observed in the within-UIP cellular dynamics and pseudotime trajectories (Figures 3C and 3D). By acquiring antioxidant and detoxification pathways together with elevated Wnt ligand production, LUSC3 has specifically adapted to the oxidative stress-rich fibrotic environment of UIP and become selectively enriched there, which highlight its emergence as a further-evolved, stress-resilient lineage in LUSC. Figure S4C shows stacked bar plots of Cell2location-inferred cell type proportions. Figures S4D and S4E demonstrate the accuracy of our workflow by showing deconvolution of tumor-stroma interface ROIs with and without compartment segmentation, using Pan-CK expression.

Spatial expression of lineage markers across regions (Figure 4F) confirmed the cell type distributions inferred by deconvolution. Both metaplastic basal cell markers (*KRT5* and *KRT17*)^21^ and club cell markers (*SCGB1A1* and *SCGB3A1*) were significantly upregulated in In-F relative to Out-N. Spatial expression of scRNA-seq-derived LUSC3-specific DEGs revealed that Wnt ligands and ABC transporters tended to be more highly expressed in In-T than in Out-T ROIs, with several genes showing significant increases in their expression (Figure 4G). The KEAP1-NRF2 pathway targets also trended higher in In-T but did not reach statistical significance, likely reflecting the resolution limits of “local-bulk” spatial transcriptomics (Figure S4F). Interestingly, Wnt ligands were upregulated not only in In-T versus Out-T but also in In-F versus Out-N, and *WNT3* was significantly upregulated in both comparisons.

To corroborate these transcriptomic findings at the protein level, we performed immunohistochemistry (IHC) targeting nuclear factor erythroid 2-related factor 2 (NRF2) and WNT3 (Figure 4H). We focused our evaluation on the tumor periphery, hypothesizing that tumor cells at the boundary are most directly exposed to the high oxidative stress characteristic of the UIP lung environment. In specimens from In-UIP patients, we observed a distinct regional co-expression of *WNT3* and *NRF2* specifically in tumor cells at the boundary facing the fibrotic background. In contrast, while tumors from Out-UIP patients exhibited mild *WNT3* expression at the boundary, they showed negligible *NRF2* expression in the same regions. These histological observations support our hypothesis that the LUSC3 subclone acquires NRF2-mediated oxidative stress tolerance to adapt to and survive within the hostile, pro-oxidant environment of UIP. Leveraging the complementary strengths of single-cell and spatial transcriptomics coupled with histological validation, we revealed a coordinated program of Wnt ligand production and antioxidant detoxification specific to the LUSC3 subclone within the UIP-associated fibrotic niche.

### Expansion of basal cells validated in external fibrosing ILD cohorts

**Figure 5:**
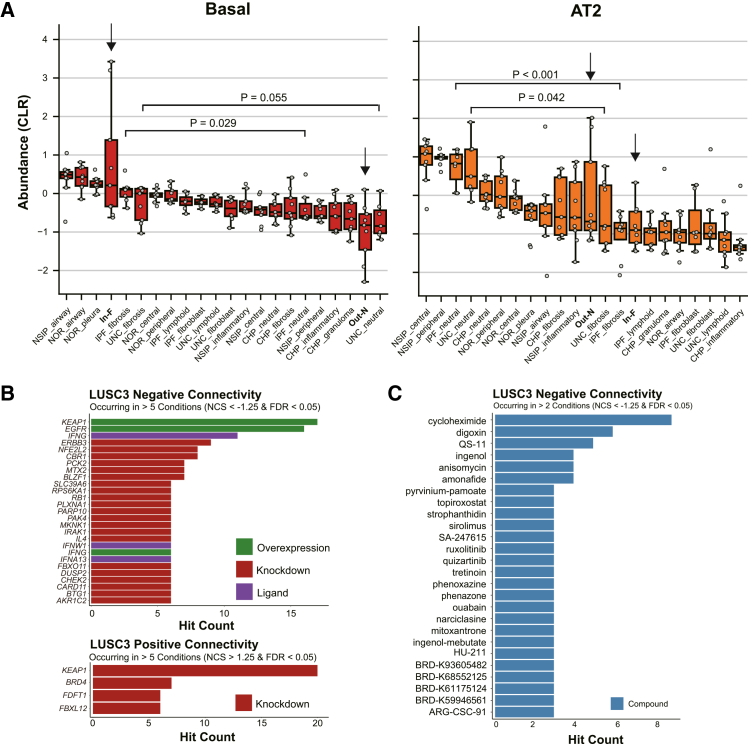
External validation of basal cell expansion and AT2 depletion in fibrosing ILDs, and connectivity analysis of the LUSC3 signature (A) Basal and AT2 cell abundances across lung microenvironments. Boxplots display the CLR-transformed abundances of basal and AT2 cells for histological microenvironment present in our non-tumor ROIs and in the external ILD dataset (GSE255174). Categories are ordered by the median value within each boxplot. Statistical test (Wilcoxon rank-sum test) results are shown between the fibrotic and uninvolved regions in IPF and UNC. Black arrows mark the two classes derived from our study. Individual gray dots represent each ROI; boxes denote the median and inter-quartile range; whiskers extend to 1.5× IQR. Abbreviations: IPF, idiopathic pulmonary fibrosis; NSIP, non-specific interstitial pneumonia; CHP, chronic hypersensitivity pneumonitis; UNC, unclassifiable fibrosing interstitial lung diseases; NOR, normal control; fibrosis, fibrosis area; fibroblast, fibroblastic foci; lymphoid, lymphoid aggregate area; neutral, fibrosis-uninvolved area; peripheral, peripheral lung fibrosis; central, central lung fibrosis; inflammatory, inflammatory area; airway, bronchial area; granuloma, granuloma area; pleura, pleural region. (B) Genetic perturbations with strong negative or positive connectivity to the LUSC3 signature. Bar plots display the number of independent conditions (perturbagen × cell line × dose × time), in which each perturbation met |NCS| > 1.25 and FDR < 0.05. Only perturbations observed in >5 conditions are displayed. (C) Top compounds reversing the LUSC3 signature (drug-repositioning candidates). Bars indicate the number of supporting conditions per compound. Only compounds showing significant negative connectivity (NCS < −1.25 and FDR < 0.05) in >2 experimental conditions are plotted.

Applying our cellular deconvolution workflow to the public GeoMx dataset GSE255174,^44^ we deconvoluted 170 ROIs spanning four fibrosing ILD diagnoses (IPF, non-specific interstitial pneumonia [NSIP], chronic hypersensitivity pneumonitis [CHP], and unclassifiable fibrosing interstitial lung diseases [UNC]) and normal controls. Figure 5A combines CLR-transformed basal and AT2 cell abundances from our non-tumor ROIs and from the public dataset across defined histological microenvironments. In IPF, both basal cells and AT2 cells showed significant differences in abundance between fibrotic and uninvolved alveolar regions. Basal cells were significantly enriched in fibrotic regions (*p* = 0.029), whereas AT2 cells were significantly depleted (*p* < 0.001). Interestingly, unlike basal cells, secretory lineages such as club and goblet cells did not show enrichment in the fibrotic regions of this external cohort (Figure S5A). Regardless of the variability in secretory cell detection, basal cells were consistently expanded across datasets, which highlighted them as the most robust and conserved feature of the fibrotic niche. NSIP and CHP, by contrast, displayed no consistent trends in these epithelial subsets between fibrotic and uninvolved regions. However, UNC exhibited a significant depletion of AT2 cells (*p* = 0.042) and a marked accumulation of basal cells (*p* = 0.055) in fibrotic regions, mirroring the pattern observed in IPF (Figure 5A). This suggests that the expansion of AT2-derived metaplastic basal cells may be a shared feature of fibrotic lung diseases characterized by severe alveolar injury and remodeling reported by Kathiriya et al.^21^ Our single-cell analysis identified these metaplastic basal cells as the likely cell of origin for LUSC arising in the fibrotic lesion, suggesting that their selective expansion in IPF underlies the increased LUSC incidence in these patients.^45^

### Connectivity analysis identifies the KEAP1-NRF2 axis and Wnt signaling as vulnerabilities in LUSC3

Next, we generated the LUSC3-up and downregulated signatures by selecting DEGs from our scRNA-seq comparison of LUSC3 versus the other malignant subclones (Table S2). We then queried these signatures, using the CLUE web portal under the “L1000 Query” workflow, filtering for recurrent significant hits, which revealed that the overexpression of *KEAP1* produced the strongest negative connectivity, whereas *KEAP1* knockdown ranked highest for positive connectivity (Figure 5B). Overexpression of *NFE2L2* also scored among the top negative hits, reinforcing the central role of the KEAP1-NRF2 axis. Phase 1 detoxification enzymes (*AKR1C2* and *CBR1*) also emerged as negative connectivity perturbations, reflecting their contribution to the LUSC3 detoxification program. These results nominate these genes as key regulators whose modulation reverses the LUSC3 program, highlighting them as potential targets for further therapeutic investigation. In addition to genetic perturbations, we analyzed chemical perturbagens to identify potential therapeutic compounds (Figure 5C). Notably, pyrvinium pamoate (PP), a known Wnt/β-catenin signaling pathway inhibitor,^46^ emerged as a top hit with a negative connectivity score, suggesting its potential to reverse the LUSC3 transcriptomic state. Other significant hits included digoxin, a cardiac glycoside known to inhibit NRF2,^47^ and several agents implicated in fibrosis modulation. These results nominate these pathways and compounds as key targets for reversing the LUSC3 program.

### Functional validation of the LUSC3-associated antioxidant phenotype and the role of Wnt signaling

**Figure 6:**
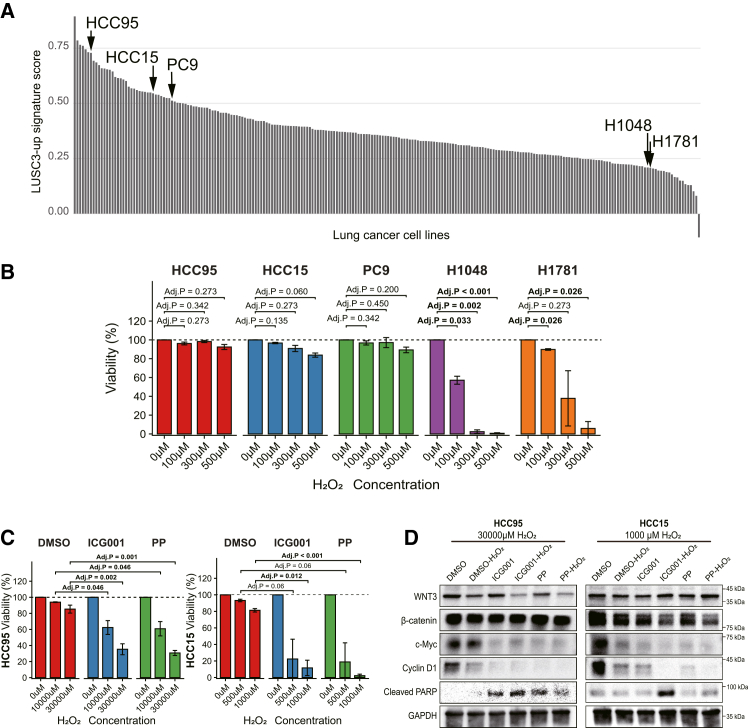
Functional validation of the LUSC3-associated antioxidant phenotype (A) Bar plot showing the single-sample gene set enrichment analysis (ssGSEA) scores for the LUSC3-up signature across human lung cancer cell lines. (B) Evaluation of oxidative stress resistance. The indicated lung cancer cell lines were treated with increasing concentrations of H_2_O_2_ (0–500 μM) for 8 h in low-serum conditions (0.5% FBS). Cell viability was assessed by crystal violet staining, quantified, and then normalized relative to the control (0 μM H_2_O_2_). Data represent the mean ± SD of independent replicates (*n* = 3). Statistical significance was determined against control (0 μM H_2_O_2_), using Student’s *t* test, followed by the Holm-Bonferroni correction. (C) Impact of Wnt/β-catenin signaling inhibition on oxidative stress resistance. HCC95 and HCC15 cells were pre-treated with vehicle (DMSO; 0.1%), ICG001 (10 μM), or pyrvinium pamoate (PP; 150 nM) in 0.5% FBS medium, followed by co-treatment with the indicated concentrations of H_2_O_2_. Cell viability was assessed by crystal violet staining, quantified, and normalized relative to the control (0 μM H_2_O_2_). Data represent the mean ± SD of independent replicates (*n* = 3). Statistical significance was determined against DMSO at each H_2_O_2_ concentration by using Student’s *t* test followed by the Holm-Bonferroni correction. (D) Western blot analysis of Wnt signaling and apoptosis markers under oxidative stress. HCC95 and HCC15 cells were pre-treated with vehicle, ICG001, or PP in 0.5% FBS medium, followed by exposure to H_2_O_2_ (30,000 μM for HCC95; 1,000 μM for HCC15). GAPDH served as a loading control. Full uncropped images are provided in Figure S6A.

To experimentally validate the functional advantage of the LUSC3 subclone, we first sought to identify representative cell line models that recapitulate the LUSC3 transcriptomic profile. We performed single-sample gene set enrichment analysis (ssGSEA) on RNA-seq data from 232 lung cancer cell lines, using the LUSC3-up signature (Table S2). Notably, HCC15, a cell line known to harbor *KEAP1* mutation,^48^ exhibited one of the highest signature scores. On the basis of this result, we selected HCC15, HCC95, and PC9 as LUSC3 signature-high models, and NCI-H1048 (H1048) and NCI-H1781 (H1781) as LUSC3 signature-low controls for further *in vitro* experiments (Figure 6A).

Next, to validate whether the LUSC3 transcriptomic signature confers a functional survival advantage under oxidative stress, we examined the cellular sensitivity to H_2_O_2_ in the selected cell line models. The cell lines were exposed to increasing concentrations of H_2_O_2_ (0–500 μM) for 8 h, and viability was assessed. Consistent with their high signature scores, the LUSC3-high cell lines (HCC15, HCC95, and PC-9) exhibited significantly higher resistance to oxidative injury, maintaining viability even at higher concentrations (Figure 6B). In contrast, the LUSC3-low cell lines (H1048 and H1781) were highly sensitive, showing a marked reduction in viability in a dose-dependent manner. These results indicated that LUSC3 has tolerance to oxidative stress.

To further investigate the contribution of Wnt signaling to this stress-adapted phenotype, we validated the pathway’s functional relevance. Given that our transcriptomic analysis revealed upregulation of Wnt ligands in LUSC3 and the connectivity analysis identified the Wnt inhibitor PP as a therapeutic candidate (Figure 5C), we hypothesized that this pathway supports the subclone’s oxidative stress tolerance. To test this, we performed perturbation experiments using LUSC-derived cell lines with high LUSC3 signature scores (HCC95 and HCC15). We targeted the Wnt/β-catenin pathway by using PP^46^ and ICG001, a distinct small-molecule inhibitor that inhibits Wnt/β-catenin signaling by antagonizing the β-catenin-CBP interaction.^49^ The cell lines were pre-treated with these inhibitors or vehicle control, followed by exposure to oxidative stress induced by high concentrations of H_2_O_2_. While HCC95 and HCC15 cells retained substantial viability under oxidative injury, treatment with ICG001 (10 μM) or PP (150 nM) significantly restored their sensitivity to H_2_O_2_-induced cytotoxicity (Figure 6C). These functional data suggest that Wnt/β-catenin signaling plays a supportive role in maintaining the oxidative stress tolerance characteristic of the LUSC3 subclone.

To explore the molecular mechanism underlying this enhanced sensitivity, we performed western blot analysis to assess the status of Wnt signaling and apoptotic markers (Figure 6D). In both HCC95 and HCC15 cell lines, treatment with ICG001 or PP resulted in a consistent downregulation of Wnt/β-catenin downstream targets, particularly c-Myc and cyclin D1. Notably, in HCC15 cells, the combination treatment led to the near-complete abrogation of c-Myc and cyclin D1 expression, accompanied by a marked increase in cleaved poly(ADP-ribose) polymerase (PARP) levels, indicating the induction of apoptosis. Regarding upstream proteins (WNT3 and β-catenin), while suppressive trends were observed under combination treatment, the patterns were variable; WNT3 reduction was prominent in HCC95 cells, whereas β-catenin reduction was more evident in HCC15 cells. This variation likely reflects the cell line-specific characteristics or differences in the oxidative stress conditions applied (30,000 μM vs. 1,000 μM H_2_O_2_). Nevertheless, the consistent suppression of downstream targets and induction of apoptosis strongly suggest that the Wnt/β-catenin pathway plays a critical role in protecting LUSC3-like cells from oxidative stress-induced cell death.

## Discussion

In this study, we performed a multi-omics analysis to compare LUSC tumors arising within versus outside UIP-affected areas in patients with IPF, aiming to clarify the molecular pathological features of LUSC associated with UIP. A unique aspect of our methodology involves establishing reliable cell signatures by visualizing carcinogenesis and tumor subclone differentiation within fibrotic lesions, using trajectory analysis, followed by their rigorous integration with spatial transcriptomics via deconvolution. Unlike standard histological, bulk molecular, or single-modal omics approaches, this strategy enabled us to precisely map the distinct cell populations to the fibrotic niche and unravel their specific adaptation mechanisms. Specifically, in the non-neoplastic regions of UIP lungs, we observed an increase in metaplastic epithelial cells—such as basal cells—and a corresponding decrease in AT2 and AT1 cells. In healthy lungs, alveoli are lined by AT1 and AT2 cells, while basal cells are confined to the airways. In contrast, IPF lungs exhibit the accumulation of basal cells in the alveolar space, where they replace resident alveolar epithelial cells and contribute to the formation of pathologic honeycomb cysts.^17^^,^^43^^,^^50^ Regarding the origin of these basal-like cells, two main hypotheses have been proposed: (1) the migration of resident basal cells from the airways—a process recently shown to be modulated by the fibrotic extracellular matrix,^50^^,^^51^^,^^52^^,^^53^ and (2) the transdifferentiation from AT2 cells.^20^^,^^21^^,^^43^^,^^54^ Our multi-omics analysis provides compelling evidence supporting the latter in the context of UIP.

We demonstrated that basal cells in the In-F region specifically express metaplastic markers such as *KRT5* and *MMP7*, a profile identical to the “KRT5^+^ metaplastic basal cells” derived from AT2 cells reported in severe alveolar injury.^21^ Furthermore, trajectory analysis revealed a continuous lineage transition from AT2 cells to metaplastic basal cells and subsequently to malignant clones in In-T. This contrasts with the disconnected lineages observed in Out-T, which likely originate from resident basal cells. Collectively, these findings suggest that LUSC tumors arising within UIP regions originate from AT2-derived metaplastic basal cells, linking the regenerative failure of AT2 cells directly to carcinogenesis.

Our findings were further supported by external validation in a larger cohort of fibrosing ILDs. Although bronchiolization—characterized by the ectopic appearance of airway epithelial cells including secretory lineages—is a hallmark of IPF,^55^^,^^56^ our external validation cohort did not show a consistent enrichment of club and goblet cells. This discrepancy likely reflects the resolution limits of spatial deconvolution, particularly given the substantial transcriptional overlap and phenotypic plasticity shared among intermediate secretory and alveolar lineages in the remodeling lung.^20^^,^^42^^,^^43^ However, the expansion of basal cells was consistently observed across all IPF (and UNC) cohorts (our data and GSE255174), reinforcing the hypothesis that AT2-derived metaplastic basal cells are the central pathogenic drivers in the fibrotic niche, distinct from a general airway ingrowth.

Interestingly, LUSC tumors arising within UIP regions showed a strong association with transcriptional profiles, characteristic of metaplastic basal cells, supporting the hypothesis that AT2-derived metaplastic basal cells may be the cell of origin for these tumors. Moreover, these tumors exhibited enrichment in pathways related to Wnt ligand biogenesis, with notable upregulation of *WNT3*. Importantly, elevated expression of *WNT3* and *WNT9A* was also detected in the non-tumor UIP tissue of patients with LUSC arising within UIP, suggesting activation of Wnt signaling in the surrounding microenvironment. The Wnt signaling cascade is broadly categorized into the canonical (β-catenin-dependent) and non-canonical pathways, including Wnt/planar cell polarity and Wnt/Ca^2+^ signaling.^57^ Wnt ligands regulate pulmonary fibrosis by influencing epithelial injury responses, fibroblast activation, and inflammation through both canonical and non-canonical pathways.^58^^,^^59^^,^^60^ Specifically, canonical *WNT3a* promotes fibroblast-to-myofibroblast differentiation, a key step in fibrosis, and induces the expression of non-canonical ligands such as *WNT5a* and *WNT11*.^60^ While *WNT3a* has been extensively studied in the context of fibrosis, reports on *WNT3* and *WNT9a* remain limited. Nonetheless, *WNT3* has been implicated in tumorigenesis across multiple malignancies, including liver, gastric, and colorectal cancers, via activation of the canonical Wnt pathway.^61^ In lung cancer, *WNT3* expression is significantly higher than in normal tissue and positively correlates with oncogenic markers such as c-Myc, survivin, and Ki-67.^62^ Notably, *WNT3* expression is higher in LUSC than in LUAD, and elevated levels are associated with greater invasiveness of NSCLC.^62^ Given that UIP typically precedes carcinogenesis, we posit that the fibrotic niche drives the selection of LUSC3. However, we cannot rule out the complementary hypothesis that LUSC3 actively remodels its microenvironment. The observed upregulation of Wnt ligands in LUSC3 suggests that the tumor may actively promote fibrosis and oxidative stress, thereby creating a niche that favors its own survival.

LUSC arising within UIP also showed enrichment of the NRF2-related antioxidant pathways, including the KEAP1-NFE2L2 axis. Oxidative stress is known to be pronounced within fibrotic lesions of IPF, contributing to progressive fibrosis, alveolar epithelial cell injury, immune cell activation, and fibroblast differentiation.^63^^,^^64^^,^^65^ The NRF2 pathway has a dual role in lung cancer: initially, it plays a protective role by mitigating oxidative stress and DNA damage, but later it plays an oncogenic role by promoting tumor progression, therapy resistance, and metastasis.^66^^,^^67^^,^^68^^,^^69^ Aberrant activation of NRF2, often due to *KEAP1* or *NFE2L2* mutations, enhances cancer cell survival and contributes to resistance against chemotherapy, radiotherapy, and immunotherapy.^69^^,^^70^ Our functional validation demonstrated that LUSC3-like cells exhibit robust resistance to H_2_O_2_-induced oxidative injury (Figure 6B), directly supporting the notion that NRF2 activation confers a survival advantage in this hostile environment. Moreover, NRF2 can facilitate immune evasion by upregulating immunosuppressive enzymes such as KYNU, which promote regulatory T cell function and correlate with a poor prognosis.^71^ The prominent NRF2-related responses observed in LUSC3 likely reflect adaptation to the oxidative and inflammatory milieu of the fibrotic lung microenvironment. Collectively, these findings suggest that NRF2 activation in LUSC3 serves a dual function: protecting against the oxidative environment of fibrosis, and potentially facilitating immune evasion.

Recent genomic studies further support a molecular link between IPF and LUSC. Frequent alterations in *SETD2* and *NFE2L2*, as well as *MYC* amplification, have been reported in patients with LUSC and IPF.^72^ Notably, *SETD2* mutations were associated with poor outcomes. Additionally, germline mutations in surfactant proteins SFTPA1 and SFTPA2 have been implicated in the development of both idiopathic interstitial pneumonia and lung cancer.^73^^,^^74^ In our cohort, we did not detect any somatic mutations typical to these reported IPF-associated lung cancers (Figure S5B).

The identification of several FDA-approved agents and bioactive compounds as potential inhibitors of the LUSC3 signature supports the feasibility of a drug-repositioning strategy (Figure 5C). PP, which we validated *in vitro*, is originally an antihelmintic but is increasingly being recognized as a potent Wnt pathway inhibitor. It has been shown to inhibit breast cancer stem cell self-renewal^75^ and enhance glioblastoma sensitivity to temozolomide via Wnt/β-catenin-mediated suppression.^76^ Our functional data demonstrated that PP restores the sensitivity of LUSC3-like cell lines to oxidative stress, suggesting that it targets Wnt-dependent survival mechanisms. Given the dominant role of the KEAP1-NRF2 axis in oxidative stress tolerance, Wnt signaling likely functions as a supportive or complementary mechanism to reinforce this resistance. This concept is consistent with emerging evidence linking Wnt/β-catenin signaling to the regulation of cellular metabolism and redox homeostasis.^77^ Specifically, Wnt signaling has been reported to confer resistance to ferroptosis—an oxidative, iron-dependent form of cell death—by regulating antioxidant enzymes such as GPX4.^78^ This implies that the characteristic features of LUSC3, namely high Wnt ligand expression and oxidative stress tolerance, are not independent phenomena but are mechanistically coupled, with Wnt signaling serving as a supportive mechanism to reinforce the antioxidant defense. In addition to PP, several other agents showed recurrent significant hits. Digoxin, a cardiac glycoside, has been shown to inhibit NRF2 signaling and resensitize gemcitabine-resistant pancreatic cancer cells to chemotherapy.^47^ Anisomycin, a protein synthesis inhibitor, has been shown to selectively suppress leukemia cells by inhibiting Wnt/β-catenin signaling.^79^ Sirolimus and ruxolitinib both have demonstrated antifibrotic activity. Sirolimus reduced circulating fibrocytes in patients with IPF in a randomized controlled crossover trial^80^ and maintained local antifibrotic effects during wound healing via controlled delivery.^81^ Ruxolitinib suppressed fibrogenic signaling in preclinical models of systemic sclerosis and liver fibrosis.^82^^,^^83^ These findings support the rationale for repositioning selected compounds as add-on therapies to target oxidative stress-tolerant subclones in UIP-associated LUSC.

Taken together, our findings suggest that AT2-derived metaplastic basal cells serve as the cellular origin of LUSC in the context of UIP and that the activation of the Wnt pathway, mainly *WNT3*, may play an important role in linking fibrosis and tumorigenesis.

### Limitations of the study

A major limitation of this study is the small sample size (*n* = 2 patients; 4 samples) employed for the initial scRNA-seq analysis. While this approach allowed for high-resolution characterization of the transcriptomic landscape, we acknowledge that the identified subclonal heterogeneity and trajectory inferences are derived from a limited number of patients. Consequently, there remains a risk that some observed characteristics could reflect patient-specific phenomena. We strove to mitigate this concern by validating key findings—especially the LUSC3 phenotype and its association with the fibrotic niche—using spatial transcriptomics in a larger cohort (*n* = 6 patients; totaling 52 ROIs); however, future large-scale single-cell profiling is essential to fully establish the robustness of these classifications. Second, because all patients included in the study were considered operable, it is evident that the cohort does not include patients with more advanced or inoperable interstitial pneumonia. Nevertheless, our data demonstrate that even in such operable cases without severe IPF, AT2-derived metaplastic basal cells and aberrant secretory lineages were already expanded in fibrotic lung regions. Third, all patients in both groups had a heavy smoking history. While the limited sample size precluded multivariate statistical adjustments, the shared heavy-smoking background limits our ability to assess the independent effects of smoking, but it also suggests that the observed differences between groups are less likely to be attributable to differences in smoking exposure. Fourth, although we validated our findings in an external cohort, discrepancies in secretory cell detection highlight the potential resolution limits of spatial deconvolution in distinguishing closely related intermediate cell states within the complex fibrotic niche. Finally, regarding mechanistic validation, while we demonstrated the functional dependence of LUSC3-like cells on Wnt signaling by using pharmacological inhibitors, we did not perform genetic manipulation of specific ligands such as *WNT3*. Thus, the specific causal contribution of *WNT3* (and other canonical Wnt ligands) remains to be defined in future studies. Despite these limitations, this is the first study to integrate scRNA-seq and spatial transcriptomics in IPF-associated lung cancer and provides important insights into the molecular pathogenesis of LUSC arising in the context of IPF.

## Resource availability

### Lead contact

Request for further information and resources should be directed to and will be fulfilled by the lead contact, Kazuhiko Shien (k.shien@okayama-u.ac.jp).

### Materials availability

This study did not generate new unique reagents.

### Data and code availability

• The scRNA-seq data are available from the Gene Expression Omnibus (GEO) under accession code GEO: GSE305872. The spatial transcriptomics data are available from GEO under accession code GEO: GSE305762. Other data reported in this paper will be shared by the lead contact upon request.
• This paper does not report original code or analysis tool.
• Any additional information required to reanalyze the data reported in this paper is available from the lead contact upon request.

## Acknowledgments

This work was supported by a Grant-in-Aid for Scientific Research from the 10.13039/501100001691Japan Society for the Promotion of Science (JSPS KAKENHI grant numbers JP23K27687 and JP23K18317 to K. Shien, JP23K27688 to S. Tomida, and JP23K24421 to S. Toyooka). We thank Yumiko Kawase, Yayoi Kubota, Yukari Kawai (Center for Comprehensive Genomic Medicine, 10.13039/100031296Okayama University Hospital, Okayama, Japan), and Fumiko Isobe (Department of Thoracic, Breast and Endocrinological Surgery, 10.13039/501100012330Okayama University Graduate School of Medicine, Dentistry and Pharmaceutical Sciences, Okayama, Japan) for their technical supports. Graphical abstract and Figure 4A were created in BioRender. A.Mat. https://BioRender.com/yh3ihep and https://BioRender.com/zqg8t3v.

## Author contributions

Conceptualization, A. Matsuoka, K. Shien, M. Ohki, and S. Tomida; data curation, A. Matsuoka; formal analysis, A. Matsuoka; investigation, A. Matsuoka, M. Ohki, and H.I.; methodology, A. Matsuoka, K. Shien, and S. Tomida; validation, A. Matsuoka and S. Tomida; visualization, A. Matsuoka; writing – original draft, A. Matsuoka and K. Shien; writing – review & editing, A. Matsuoka, K. Shien, S. Tomida, D.E., and S. Toyooka; resources, H.T., K.H., R. Fujiwara, K.I., S.M., R. Fujii, A. Mimata, K.O., R.Y., M.Y., Y.F., H.Y., K.N., S. Tanaka, K. Suzawa, K.M., M. Okazaki, and S.S.; supervision, K. Shien, S. Tomida, and S. Toyooka; funding acquisition, K. Shien, S. Tomida, and S. Toyooka; project administration, A. Matsuoka, K. Shien, and S. Toyooka.

## Declaration of interests

The authors declare no competing interests.

## Declaration of generative AI and AI-assisted technologies in the writing process

During the preparation of this work, the authors used Gemini (Google) in order to improve the readability and language of the manuscript. After using this tool, the authors reviewed and edited the content as needed and take full responsibility for the content of the published article.

## STAR★Methods

### Key resources table

**Table:** 

REAGENT or RESOURCE	SOURCE	IDENTIFIER
**Antibodies**		

CD68 Antibody	Santa Cruz	Cat# sc-20060 AF647; RRID: AB_627158
NRF2 Antibody	Abcam	Cat# ab62352; RRID: AB_944418
WNT3 Antibody	Proteintech	Cat# 28156-1-AP; RRID: AB_3669649
β-catenin Antibody	Cell Signaling Technology	Cat# 8480; RRID: AB_11127855
c-Myc Antibody	Cell Signaling Technology	Cat# 13987; RRID: AB_2631168
Cyclin D1 Antibody	Cell Signaling Technology	Cat# 55506; RRID: AB_2827374
Cleaved PARP Antibody	Cell Signaling Technology	Cat# 5625; RRID: AB_10699459
GAPDH Antibody	Cell Signaling Technology	Cat# 2118; RRID: AB_561053

**Biological samples**		

Human lung squamous cell carcinoma resected samples	Okayama University Hospital	https://www.okayama-u.ac.jp/user/hospital/en/index.html

**Chemicals, peptides, and recombinant proteins**		

SPRIselect	Beckman Coulter	Cat# B23318
BOND Wash Solution 10×Concentrate	Leica Biosystems	Cat# AR9590
20X SSC Buffer	Sigma-Aldrich	Cat# S6639
AMPure XP	Beckman Coulter	Cat# A63880
Antigen Retrieval Solution pH 9	Nichirei Bioscience	Cat# 415201
Histofine Simple Stain MAX-PO (MULTI)	Nichirei Bioscience	Cat# 424154
Histofine DAB Substrate Kit	Nichirei Bioscience	Cat# 425011
H_2_O_2_	Sigma-Aldrich	Cat# 13-1910
ICG001	Apexbio	Cat# A8217
Pyrvinium pamoate	Sigma-Aldrich	Cat# P0027
Phosphatase Inhibitor Cocktail 2	Sigma-Aldrich	Cat# P5726
Phosphatase Inhibitor Cocktail 3	Sigma-Aldrich	Cat# P0044
cOmplete Mini Protease Inhibitor Cocktail	Roche	Cat# 11836153001
Anti-rabbit IgG, HRP-linked Antibody	Cell Signaling Technology	Cat# 7074
Amersham ECL Prime Western Blotting Detection Reagent	Cytiva	Cat# RPN2232

**Critical commercial assays**		

Chromium Next GEM Single Cell Fixed RNA Sample Preparation Kit	10x Genomics	Cat# 1000414
Chromium Fixed RNA Kit, Human Transcriptome	10x Genomics	Cat# 1000475
Chromium Next GEM Chip Q Single Cell Kit	10x Genomics	Cat# 1000422
Dual Index Kit TS Set A	10x Genomics	Cat# 1000251
AllPrep DNA/RNA Mini Kit	Qiagen	Cat# 80204
SureSelect Human All Exon V6 kit	Agilent Technologies	Cat# 5190-8865
BOND Enzyme Pretreatment Kit	Leica Biosystems	Cat# AR9551
GeoMx Whole Transcriptome Atlas Human RNA for Illumina Systems	NanoString Technologies	Cat# GMX-RNA-NGS-HuWTA-4
GeoMx Solid Tumor TME Morphology Kit Human RNA Compatible	NanoString Technologies	Cat# GMX-RNA-MORPH-HST-12
GeoMx Seq Code Pack: G & H Compatible with Illumina Systems	NanoString Technologies	Cat# GMX-NGS-SEQ-GH

**Experimental models: cell lines**		

HCC15	Adi F. Gazdar	University of Texas Southwestern Medical Center
HCC95	Adi F. Gazdar	University of Texas Southwestern Medical Center
PC9	RIKEN BRC	RCB4455
NCI-H1048	ATCC	CRL-5853
NCI-H1781	ATCC	CRL-5894

**Deposited data**		

Human reference genome NCBI build 38, GRCh38	Genome Reference Consortium	http://www.ncbi.nlm.nih.gov/projects/genome/assembly/grc/human
C2 gene set (Human MsigDB v2024.1)	Subramanian et al.^84^	https://www.gsea-msigdb.org/gsea/msigdb/index.jsp
Known INDEL variants	Broad Institute	https://storage.googleapis.com/gcp-public-data--broad-references/hg38/v0/Homo_sapiens_assembly38.known_indels.vcf.gz
Panel of Normal	Broad Institute	https://storage.googleapis.com/gatk-best-practices/somatic-hg38/1000g_pon.hg38.vcf.gz
Germline resource	Broad Institute	https://storage.googleapis.com/gatk-best-practices/somatic-hg38/af-only-gnomad.hg38.vcf.gz
ToMMo 38KJPN-SNV/INDEL Allele Frequency Panel (v20220929)	Tohoku Medical Megabank Organization	https://jmorp.megabank.tohoku.ac.jp/docs/guide-ja/dataset_detail/tommo-38kjpn_snvindel
GeoMx WTA dataset of fibrosing ILD	Kim et al.^44^	GEO: GSE255174
Transcript expression levels (nTPM values) for lung cancer cell lines (v25.0)^85^	The Human Protein Atlas	https://www.proteinatlas.org/humanproteome/cell+line/data#cell_lines
Our scRNA-seq dataset	This paper	GEO: GSE305872
Our GeoMx spatial transcriptomics dataset	This paper	GEO: GSE305762

**Software and algorithms**		

Cell Ranger (v8.0.1)	10x Genomics	https://www.10xgenomics.com/support/software/cell-ranger/downloads
scanpy (v1.10.4)	Wolf et al.^17^	https://github.com/scverse/scanpy
CellTypist (v1.6.3)	Xu et al.^18^	https://github.com/Teichlab/celltypist
scvi-tools (v1.3.0)	Gayoso et al.^86^	https://github.com/scverse/scvi-tools
inferCNV (v1.3.3)	Broad Institute	https://github.com/broadinstitute/infercnv
GSEApy (v1.1.7)	Zhuoqing et al.^87^	https://github.com/zqfang/GSEApy
CellPhoneDB (v5.0.1)	Troulé et al.^36^	https://github.com/ventolab/CellphoneDB
ktplots (v2.4.0)	Troulé et al.^36^	https://github.com/zktuong/ktplots
scTour (v1.0.0)	Li et al.^39^	https://github.com/LiQian-XC/sctour
Trimmomatic (v0.39)	Bolger et al.^88^	https://github.com/usadellab/Trimmomatic
BWA (v0.7.17-r1188)	Li et al.^89^	https://github.com/lh3/bwa
Samtools (v1.13)	Li et al.^90^	https://github.com/samtools/samtools
GATK (v4.5.0.0) MarkDuplicates (Picard)	Broad Institute	https://gatk.broadinstitute.org/hc/en-us/articles/21905036102043-MarkDuplicates-Picard
GATK (v4.5.0.0) BaseRecalibrator	Broad Institute	https://gatk.broadinstitute.org/hc/en-us/articles/21905050792603-BaseRecalibrator
GATK (v4.5.0.0) ApplyBQSR	Broad Institute	https://gatk.broadinstitute.org/hc/en-us/articles/21905038144155-ApplyBQSR
GATK (v4.5.0.0) Mutect2	Broad Institute	https://gatk.broadinstitute.org/hc/en-us/articles/21905083931035-Mutect2
SnpEff (v5.2a)	Cingolani et al.^91^	https://pcingola.github.io/SnpEff/#snpeff
SnpSift (v5.2)	Cingolani et al.^92^	https://pcingola.github.io/SnpEff/#snpsift
vcf2maf (v1.6.22)	Kandoth et al.^93^	https://github.com/mskcc/vcf2maf
maftools (v2.2.0)	Mayakonda et al.^94^	https://github.com/PoisonAlien/maftools
VarTrix (v1.1.22)	10x Genomics	https://github.com/10XGenomics/vartrix
GeoMx NGS Pipeline (v2.3.3.10)	NanoString Technologies	https://jp.illumina.com/products/by-type/informatics-products/basespace-sequence-hub/apps/nanostring-geomxr-ngs-pipeline.html
GeomxTools (v3.8.0)	Griswold et al.^95^	https://www.bioconductor.org/packages/release/bioc/html/GeomxTools.html
Cell2location (v0.9.6)	Kleshchevnikov et al.^41^	https://github.com/BayraktarLab/cell2location
CLUE L1000 Query	Broad Institute	https://clue.io/query
GSVA (v1.52.3)	Hänzelmann et al.^96^	https://bioconductor.org/packages//release/bioc/html/GSVA.html
R (v4.4.0)	R Foundation	https://www.r-project.org/
Python (v3.10.16), (Under Windows 11)	Python Software Foundation	https://www.python.org/
Python (v3.8.20), (Under Ubuntu 22.04)	Python Software Foundation	https://www.python.org/
Fiji (ImageJ distribution)	Schindelin et al.^97^	https://fiji.sc/

**Other**		

gentleMACS Octo Dissociator	Miltenyi Biotec	N/A
Chromium Fixed RNA Profiling Reagent Kits for Multiplexed Samples (User Guide/CG000527/Rev D)	10x Genomics	https://cdn.10xgenomics.com/image/upload/v1680118519/support-documents/CG000527_Chromium_FixedRNAProfiling_MultiplexedSamples_UserGuide_Rev_D.pdf
Chromium iX	10x Genomics	N/A
NovaSeq 6000 platform	Illumina	N/A
AllPrep DNA/RNA Mini Handbook	Qiagen	https://www.qiagen.com/us/resources/resourcedetail?id=580866a6-56c6-4674-8566-2852164d8519&lang=en
NovaSeq X platform	Illumina	N/A
GeoMx DSP Automated Slide Preparation User Manual (MAN-10151-06)	NanoString Technologies	https://university.nanostring.com/geomx-dsp-automated-slide-preparation-user-manual/1209595
GeoMx DSP Instrument User Manual (MAN-10152-06)	NanoString Technologies	https://university.nanostring.com/geomx-dsp-instrument-user-manual/1163226
GeoMx DSP NGS Readout User Manual (MAN-10153-06)	NanoString Technologies	https://university.nanostring.com/geomx-dsp-ngs-readout-user-manual/1193408
BOND RXm	Leica Biosystems	N/A
GeoMx Digital Spatial Profiler (DSP)	NanoString Technologies	N/A
TC20 Automated Cell Counter	Bio-Rad	N/A
LuminoGraph II	ATTO	N/A

### Experimental model and study participant details

#### Study design and population

This retrospective study included seven Japanese patients who underwent surgical resection for primary LUSC at Okayama University Hospital. All had preoperative CT findings of UIP, suggestive of ILD, especially IPF. Inclusion required stored surgical specimens, and the study was approved by the Okayama University Clinical Research Ethics Committee (approval number: 1906-033). Tumor and adjacent non-tumor tissues were preserved as fresh-frozen samples (snap-frozen, −80°C) and as formalin-fixed paraffin-embedded (FFPE) blocks. scRNA-seq was performed on fresh-frozen samples from two patients (tumor and adjacent tissues within and outside UIP regions). Spatial transcriptomics was performed on FFPE samples from six patients (three with tumors within UIP and three outside UIP). WES was performed on frozen tumor and adjacent tissues from six patients (excluding one patient) to identify somatic mutations.

### Method details

#### Single-cell RNA sequencing: Library preparation and sequencing

For the In-UIP case, we selected a patient with extensive UIP lesions surrounding the tumor confirmed on CT, ensuring the tumor originated within the UIP region. For the Out-UIP case, we selected a patient where the tumor was located distantly from UIP lesions without co-existing emphysematous changes. Both selected cases yielded sufficient fresh frozen tissue (51–55 mg each) without necrosis. Tissue fixation and dissociation were carried out using the Chromium Next GEM Single Cell Fixed RNA Sample Preparation Kit (10x Genomics) following the manufacturer’s protocol. Briefly, minced tissues were fixed in fixation buffer for 24 hours at 4°C and dissociated using the gentleMACS Octo Dissociator (Miltenyi Biotec). Fixed single-cell suspensions were hybridized with probe barcodes using the Chromium Fixed RNA Kit, Human Transcriptome (10x Genomics). After a 19-hour hybridization at 42°C, unbound probes were removed and equal numbers of cells from each sample were pooled. Pooled cells and barcoded gel beads were co-partitioned into Gel Beads-in-Emulsions (GEMs) using the Chromium iX (10x Genomics) and the Chromium Next GEM Chip Q Single Cell Kit (10x Genomics). Following GEM generation, probe ligation and pre-amplification by PCR were performed. Amplified products were purified with SPRIselect beads (Beckman Coulter) and used to construct gene expression libraries. Sample indexing and Illumina adapter ligation were performed using the Dual Index Kit TS Set A (10x Genomics). Library quality was assessed using TapeStation (Agilent Technologies), and sequencing was performed on an Illumina NovaSeq 6000 platform (paired-end 150 bp) at Rhelixa, targeting approximately 1.6 billion reads (800 million read pairs). FASTQ files were generated from the resulting raw sequencing data.

#### Data preprocessing and primary analysis

FASTQ files were processed using cellranger multi (Cell Ranger v8.0.1, 10x Genomics) with default settings. Analysis was performed with the human reference transcriptome (GRCh38, 2024-A) and the Human Transcriptome Probe Set v1.0 provided by 10x Genomics. Gene expression matrices were analyzed using Scanpy (v1.10.4)^17^ in Python (v3.10.16) on Windows 11. Four tissue regions were analyzed from two representative patients: tumor within the UIP lesion (In-T), adjacent fibrotic tissue (In-F), tumor outside the UIP lesion (Out-T), and adjacent normal lung (Out-N). Cells with <200 or >5000 detected genes were removed to exclude low-quality cells and potential doublets. Cells with >5% mitochondrial or >1% hemoglobin gene content were also excluded. Genes expressed in fewer than 3 cells were filtered out. Final cell counts were: In-T, 9262; In-F, 6293; Out-T, 2774; Out-N, 3030. Normalization was performed using total-count normalization (target sum = 10000 counts per cell) with sc.pp.normalize_total(), followed by log1p transformation with sc.pp.log1p(). Preliminary cell type annotation was performed using CellTypist (v1.6.3)^18^ with the ‘Human_IPF_Lung.pkl’ model and majority voting. Highly variable genes (HVGs) were selected using sc.pp.highly_variable_genes() (top 2000 genes). Batch effect correction and latent representation learning were conducted using scvi-tools (v1.3.0).^86^ The AnnData object was registered using SCVI.setup_anndata() with “patient” as the batch key. The SCVI model was configured with 2 layers and 30 latent dimensions using a negative binomial likelihood and trained for 400 epochs. Graph-based clustering was performed on the SCVI latent space. A k-nearest neighbors graph (k=10) was constructed using sc.pp.neighbors(), followed by Leiden clustering (sc.tl.leiden(), resolution=1.5). UMAP was applied with sc.tl.umap() using a minimum distance of 0.45 for visualization. Final cell type annotation was manually refined based on the Leiden clusters and validated by known marker gene expression. Cells classified as malignant or cancer-associated fibroblasts (CAF) within non-tumor tissues (In-F, Out-N) were removed (In-F: Malignant 0.069%, CAF 0.042%; Out-N: Malignant 0.231%, CAF 0.298%). To support malignant cell identification, CNV inference was performed using inferCNV (v1.3.3) in R (v4.4.0), using Out-N cells as references. CNV states were inferred via hidden Markov models with denoising, and per-cell SDs were calculated. Malignant cells were subsetted and subjected to subclustering. HVGs (top 4000) were re-selected, and SCVI was re-applied using the same configuration and training parameters. Clustering was repeated with k=15 and Leiden resolution=0.25. Four malignant subclones were identified and annotated as LUSC1, LUSC2, LUSC3, and LUSC4. Annotations were transferred back to the full dataset for downstream analysis.

#### Differential expression analysis

Differential gene expression analysis between malignant subclones was performed using the sc.tl.rank_genes_groups() function in Scanpy. The Wilcoxon rank-sum test, with Benjamini-Hochberg (BH) correction was used to compare gene expression between each cluster and the rest.

#### Over representation analysis

To identify enriched biological pathways and processes within each malignant subclone, ORA was performed using the enrichr() function in GSEApy (v1.1.7)^87^ DEGs identified as described above were used as input. Each subclone was analyzed against the C2 gene set collection from the Molecular Signatures Database (MSigDB v2024.1).^84^^,^^98^ The background gene list included all genes expressed in the dataset.

#### Ligand–receptor interaction analysis

Cell–cell communication analysis was performed using CellPhoneDB (v5.0.1)^36^ with the statistical inference method. The pipeline was executed in Python (v3.8.20) on a Linux environment (Ubuntu 22.04). Gene expression matrices and cell type annotations were used as input. Ligand–receptor interactions were inferred using the CellPhoneDB v5 database, and statistical analysis was performed using the cpdb_statistical_analysis_method() function. Visualization of inferred interactions was carried out using the ktplots package (v2.4.0)^36^ in R.

#### PAGA graph construction and visualization

To explore the global topological structure and lineage relationships between malignant and non-malignant epithelial populations, we performed PAGA using the sc.tl.paga() function in Scanpy. The resulting graph was visualized with the sc.pl.paga() function. To infer the potential cell-of-origin of malignant cells, we incrementally increased the edge threshold parameter to exclude low-confidence connections and identify the minimal graph in which only a single non-malignant epithelial population remained connected to the malignant cluster. The final threshold was set to 0.06.

#### Trajectory analysis

Single-cell trajectory inference and pseudotime analysis were performed using scTour (v1.0.0).^39^ Tumor-derived cells, including LUSC subclones, basal cells, and AT2 cells, were extracted from each sample; AT2 cells were included as a control population representing distal alveolar epithelial cells. The top 2000 HVGs were used as input. scTour projected cells into a latent space using mean squared error (MSE) loss and numerically solved ordinary differential equations via the Euler method. Training was conducted for 400 epochs. The latent representations were computed as a weighted combination of inferred (α_z = 0.3) and predicted (α_predz = 0.7) latent variables. UMAP was used to visualize cellular clusters and pseudotime trajectories, with vector fields overlaid to indicate directionality. Vector directions were reversed post-inference according to scTour documentation to align with biological interpretation. To visualize subclone distribution along the pseudotime axis, kernel density estimates (KDEs) were computed for each LUSC subclone. KDE curves were weighted by the relative abundance of each subclone.

#### Whole-exome sequencing: Library preparation and sequencing

Tumor and adjacent non-tumor tissues from six patients were used for WES. For each sample, 20–30 mg of frozen tissue was homogenized using PowerMasher II (Nippi Inc.). Genomic DNA was extracted using the AllPrep DNA/RNA Mini Kit (Qiagen) following the manufacturer’s protocol. Exome capture and library preparation were performed using the SureSelect Human All Exon V6 kit (Agilent Technologies). Sequencing was conducted using the NovaSeq X platform (Illumina) with 150 bp paired-end reads, targeting approximately 40 million reads per sample. Library preparation and sequencing were performed at Macrogen Japan.

#### Data processing

Adapter trimming and quality filtering were performed using Trimmomatic (v0.39).^88^ Filtered reads were aligned to the GRCh38 reference genome using the BWA-MEM algorithm in BWA (v0.7.17-r1188).^89^ Aligned BAM files were sorted using Samtools (v1.13).^90^ Duplicate reads were marked using MarkDuplicates (GATK v4.5.0.0).^99^ Base quality score recalibration (BQSR) was performed using BaseRecalibrator and ApplyBQSR (GATK) with known INDEL variant information. Somatic variants were called using Mutect2 (GATK) on tumor–normal pairs, incorporating a Panel of Normals (PON) and a germline resource to reduce false positives. Variant calling was restricted to the capture regions defined by the SureSelect Human All Exon V6 target BED file. VCF files were annotated using SnpEff (v5.2a)^91^ and SnpSift (v5.2)^92^ with allele frequency data from the ToMMo 38KJPN-SNV/INDEL Allele Frequency Panel (v20220929). Variants were filtered in two steps: first, technical filters were applied (read depth <25, base quality <20, <3 alternate reads in tumor, >3 alternate reads in normal); second, variants with an allele frequency ≥0.01 in the ToMMo dataset were excluded as common variants in the general population. Filtered variants were converted to Mutation Annotation Format (MAF) files using vcf2maf (v1.6.22).^93^ Tumor mutational burden (TMB) was estimated using the tcgaCompare() function in maftools (v2.2.0),^94^ assuming a capture size of 60 Mb. Oncoplot visualization was also performed using maftools.

#### Variant-based single-cell analysis

Allele-specific expression of somatic variants was assessed at single-cell resolution using VarTrix (v1.1.22, 10x Genomics). BAM files derived from scRNA-seq (preprocessed with Cell Ranger) and processed VCF files from paired tumor-normal WES were used as inputs. Reads with mapping quality <30 were excluded. The GRCh38 reference genome and filtered cell barcodes were provided to VarTrix in coverage mode. This generated two sparse matrices per sample, indicating the number of reads supporting either the reference or alternate allele for each variant in each cell. Cells were classified as “NoCoverage,” “RefOnly,” “Multiple,” or as carrying a specific variant, based on the following criteria: cells with ≥2 alternate reads were considered variant-positive, while cells with only a single alternate read were excluded to minimize noise. The resulting matrices were integrated with the UMAP coordinates of the scRNA-seq data to visualize the spatial distribution of somatic mutations in individual cells.

#### Spatial transcriptomics: Library preparation and sequencing

Spatial transcriptomics was performed using the GeoMx DSP (NanoString Technologies) according to the manufacturer’s instructions (Automated Slide Preparation User Manual, Instrument User Manual, NGS Readout User Manual). Six FFPE surgical specimens were used. Tumor and adjacent regions (In-F or Out-N) were identified on HE-stained slides, and corresponding unstained serial sections (5 μm) were macrodissected and mounted on two slides. The BOND RXm system (Leica Biosystems) was initialized, and reagents were loaded, including BOND Wash Solution (Leica Biosystems), 10% Neutral Buffered Formalin (NBF), NBF stop buffer (prepared by combining 6.06 g Tris base and 3.75 g glycine in 500 mL DEPC-treated water), and Enzyme1 (Proteinase K) from the BOND Enzyme Pretreatment Kit (Leica Biosystems). The slides were processed for deparaffinization, antigen retrieval, and enzymatic digestion. Hybridization was performed using the GeoMx Whole Transcriptome Atlas Human RNA for Illumina Systems (NanoString Technologies), targeting human protein-coding genes. Slides were incubated overnight at 37°C in a humidified chamber. After SSC buffer (Sigma-Aldrich) washes, morphology markers were applied, including the GeoMx Solid Tumor TME Morphology Kit Human RNA Compatible (NanoString Technologies) and a CD68 antibody (Santa Cruz). ROIs were manually defined to include core tumor regions (center of tumor nests, Pan-CK highly positive with nuclear enlargement), adjacent fibrotic areas (In-F; from three In-UIP patients, characterized by relatively low Pan-CK expression, dense SYTO13 nuclear staining), and morphologically normal alveolar regions (Out-N; from three Out-UIP patients). For the tumor–stroma interface, segmentation was performed using Pan-CK expression as a marker. Pan-CK-positive regions were defined as the tumor compartment, while Pan-CK-negative regions were designated as the stromal compartment. Additionally, adjacent regions were selected without segmentation (Full ROI). In total, 52 ROIs were selected: In-F (n = 9), Out-N (n = 9), In-T [core tumor] (n = 6), Out-T [core tumor] (n = 7), tumor interface (PanCK^+^ segmentation, n = 7), stroma interface (PanCK^-^ segmentation, n = 7), and Full ROI of tumor–stroma interface (n = 7). UV light was used to release oligonucleotide tags from the tissue, which were collected into 96-well plates. The GeoMx Seq Code Pack: G & H Compatible with Illumina Systems (NanoString Technologies) was used for PCR amplification. Amplified libraries were purified using AMPure XP beads (Beckman Coulter), washed with 80% ethanol, and eluted in 10 mM Tris-HCl (pH 8.0) with 0.05% Tween-20. Library quality was assessed using TapeStation (Agilent Technologies), and sequencing was performed on an Illumina NovaSeq 6000 platform (150 bp paired-end) at a depth proportional to the total ROI area, in accordance with the manufacturer’s recommended depth.

#### Data preprocessing

FASTQ files were processed using the GeoMx NGS Pipeline (v2.3.3.10; NanoString Technologies) to generate Digital Count Conversion (DCC) files. Subsequent quality control (QC), normalization, and quantification were conducted in R using the GeomxTools (v3.8.0).^95^ For segment-level QC, ROIs were retained only if they met the following thresholds recommended in GeomxTools documentation: total reads ≥1,000, alignment rate ≥80%, stitching rate ≥80%, trimming rate ≥80%, sequencing saturation ≥50%, ≥100 nuclei per segment, and a minimum surface area of 5,000 μm^2^. All 52 ROIs in this dataset satisfied these criteria and were retained for downstream analysis. Then, probe-level QC was applied to exclude low-quality probes. Probes were globally removed if their geometric mean count across all segments fell below 10% of the target’s probe set mean or if flagged as outliers by Grubbs' test in over 20% of segments. Post-probe-level QC, data were summarized into a gene-level count matrix, where counts for each gene target with multiple probes were averaged geometrically. A Limit of Quantification (LOQ) was then calculated per segment based on the geometric mean and SD of negative-control probes. The LOQ threshold was defined as the maximum of twice the negative-control SD or a minimum value of 2 to ensure accurate quantification across segments. Subsequently, ROI segments with gene detection rates below 5% were excluded to improve data reliability. All ROIs passed this threshold and were retained. Following this, genes with detection rates below 5% across all segments were removed from the dataset to reduce noise and optimize statistical power. Finally, 13146 genes and all 52 ROIs were retained. The data were normalized using the Q3 normalization method.

#### Unsupervised analysis

Unsupervised analyses were performed using GeomxTools. The normalized gene expressions were log2-transformed. For dimensionality reduction based on overall gene expression per ROI, UMAP was performed using all genes. A 3D embedding was computed using the umap function and visualized for interactive exploration of ROI clustering patterns. Hierarchical clustering was also performed. To identify variable genes, the coefficient of variation (CV) was calculated for each gene using log2-transformed expression values, defined as CV = SD/mean. Genes in the top 25% of CV values were selected. A heatmap was generated using correlation distance and the ward.D linkage method for both genes and ROIs, with row-wise scaling applied.

#### Deconvolution of spatial transcriptomics ROIs: Preparation of scRNA-seq and GeoMx inputs

Deconvolution was performed using Cell2location (v0.9.6).^41^ Input datasets were prepared by formatting both the scRNA-seq and GeoMx expression data as AnnData objects. The scRNA-seq included all 18896 cells from two patients after preprocess, and raw UMI counts were used, in accordance with Cell2location recommendations. For spatial transcriptomics, raw digital counts from 52 quality-controlled ROIs that passed quality control in GeomxTools were combined with ROI metadata. Both matrices were restricted to 10128 gene symbols shared between the scRNA-seq and GeoMx datasets after quality filtering.

#### Estimation of reference cell-type signatures

Genes with low information content were filtered using the filter_genes() function with the following parameters: cell_count_cutoff = 3, cell_percentage_cutoff2 = 0.03, and nonz_mean_cutoff = 1.12, yielding 9308 genes. The regression model was initialized with the patient identity as a batch key and the manually curated cell-type annotation (22 categories) as the response variable. Model training was performed for 1000 epochs using a mini-batch size of 2500 cells. The default negative binomial likelihood was used. Upon convergence, 3000 posterior draws were sampled to estimate the mean expression level of each gene in each cell type. The resulting cell-type signature matrix was used as the reference.

#### Deconvolution of spatial transcriptomics ROIs

We applied the WTA model implemented in Cell2location, using the cell-type signatures trained in the previous step. ROIs from tumor regions (In-T, Out-T, and tumor–stroma interface ROIs [including segmentations and Full ROIs]) were deconvoluted using the full reference of 22 cell types, whereas adjacent non-tumor ROIs (In-F and Out-N) were analyzed using a reduced reference excluding tumor subclones (LUSC1–4) and CAFs. Because negative-control probes had been removed during preprocessing, an empty array was provided in place of negative-probe counts, and the recorded nuclei numbers were supplied as size factors. The Cell2location-WTA model was initialized with the observed ROI count matrix and the single-cell-derived signature matrix as the prior. Training was performed for 20000 epochs using the default negative binomial likelihood. Upon convergence, 3000 posterior samples per ROI were drawn to compute posterior means and 95% credible intervals for the abundance of each reference cell type. The final output matrix contained size-factor–normalized expected cell counts (cell_abundance_w_sf) for each ROI and cell type.

#### Assessment of reconstruction accuracy

To evaluate the fidelity of the deconvolution, we compared the observed GeoMx gene counts with the model-inferred reconstructed counts. Reconstructed gene expression for each ROI was calculated as the product of the estimated cell abundance, the reference cell-type signatures, and the ROI-specific detection efficiency. Pearson correlation coefficients (r) were calculated between log10-transformed observed and reconstructed counts to quantify the model fit for normal and tumor regions.

#### Normalization of cell-type counts and groupwise comparison

To allow for direct comparison of cell-type abundance across ROIs, raw cell count estimates from Cell2location were transformed using the CLR method. A small pseudocount (1 × 10^-6^) was added, each ROI was divided by its geometric mean, and the values were transformed using the natural logarithm. The resulting CLR values were used for statistical testing (Wilcoxon rank-sum test). For visualization, the mean abundance and its 95% bootstrapped confidence interval (CI) were calculated by resampling the CLR values 1000 times.

#### External validation using a public ILD spatial transcriptomics dataset

To assess the generalizability of our deconvolution pipeline, we reanalyzed a publicly available GeoMx WTA dataset (GSE255174) that profiles IPF and other interstitial lung diseases (ILDs), with 179 ROIs selected and histologically classified.^44^ Raw counts were processed using the same GeoMxTools workflow and quality control criteria described in the data preprocessing section. After filtering, 170 ROIs and 8160 genes were retained. All retained ROIs were deconvoluted using Cell2location with the same model configuration and hyperparameter settings described in the deconvolution of spatial transcriptomics ROIs section. Because the external dataset did not include tumor tissue, the reference matrix excluded the four LUSC subclones (LUSC1–4) and CAFs, as applied previously to the adjacent non-tumor ROIs (In-F and Out-N). The resulting cell abundance estimates were CLR-transformed and integrated with our dataset for comparative analyses.

#### Immunohistochemistry

IHC was performed on 4 μm FFPE human tissue sections. The sections were deparaffinized and rehydrated. Antigen retrieval was performed by heating the sections in Antigen Retrieval Solution (Nichirei Bioscience) at 95°C for 20 minutes. Endogenous peroxidase activity was blocked with 0.3% H_2_O_2_ for 10 minutes at room temperature. After washing with TBS, the sections were incubated with primary antibodies against NRF2 (1:300; Abcam) for 60 minutes or WNT3 (1:400; Proteintech) for 90 minutes at room temperature. Following washing with TBS, the sections were incubated with the secondary antibody, Histofine Simple Stain MAX-PO (MULTI; Nichirei Bioscience), for 30 minutes at room temperature. The signals were visualized using a DAB substrate kit (Nichirei Bioscience) for 5 minutes. Finally, the sections were counterstained with hematoxylin, dehydrated, and mounted.

#### Signature connectivity mapping

Connectivity analysis was performed using the CLUE L1000 Query platform. DEGs were obtained from the scRNA-seq comparison of LUSC3 to other subclones. Genes with FDR <0.05 were further filtered to retain only those valid for the L1000 analysis. From these, the top 150 upregulated and 150 downregulated genes (ranked by log2FC) were used as “Up” and “Down” signatures for the query. The L1000 query was executed using default advanced settings, including all perturbagen types, cell lines, doses, and time points. The resulting Normalized Connectivity Score (NCS) matrix and metadata were downloaded in GCT format and analyzed in R. Perturbagens were retained if they had an absolute |NCS| > 1.25 and –log10(FDR) > 1.3 (corresponding to FDR <0.05) and were recurrent across multiple experimental conditions (>5 for genes and >2 for compounds). Perturbations were categorized by mode of action: Knockdown (shRNA, CRISPR knockout, or CRISPR-mediated loss-of-function), Overexpression (cDNA-based overexpression of wild-type or mutant genes), Ligand (exogenous protein factors), or Compound (small molecule treatments).

#### Selection of lung cancer cell lines

Publicly available RNA-seq data (TPM normalized)^85^ for lung cancer cell lines were obtained from the Human Protein Atlas. The normalized values were log2-transformed, and ssGSEA was performed using GSVA (v1.52.3)^96^ with default parameters to calculate the signature score of the LUSC3-up signature (Table S2) for each cell line.

#### Cell lines culture

Five human lung cancer cell lines—HCC15, HCC95, PC9, H1781, and H1048—were used. HCC15 and HCC95 were kindly provided by Dr. Adi F. Gazdar (University of Texas Southwestern Medical Center). PC9 was purchased from RIKEN BRC cell bank. H1781 and H1048 were purchased from ATCC. All cell lines were cultured in RPMI1640 medium supplemented with 10% fetal bovine serum (FBS) and 1% Penicillin-Streptomycin at 37°C in a humidified incubator with 5% CO_2_.

#### Cell viability assay

Cell viability was assessed using crystal violet staining. Cells were counted using a TC20 Automated Cell Counter (Bio-Rad) and seeded into 6-well plates. For the H_2_O_2_ sensitivity assay, cells were seeded at the following densities: 3 × 10^5^ cells/well for PC9; 5 × 10^5^ cells/well for HCC15, HCC95, and H1781; and 7 × 10^5^ cells/well for H1048. After 24 hours of incubation, the culture medium was replaced with RPMI1640 supplemented with 0.5% FBS containing various concentrations of H_2_O_2_ (0–500 μM; Sigma-Aldrich), and the cells were incubated for 8 hours. For the combinatorial treatment assay, HCC95 (1 × 10^5^ cells/well) and HCC15 (2 × 10^5^ cells/well) were seeded. After 24 hours, the medium was changed to RPMI1640 supplemented with 0.5% FBS containing either 0.1% DMSO (vehicle control), 10 μM ICG001 (Apexbio), or 150 nM PP (Sigma-Aldrich). After 24 hours of pre-treatment, H_2_O_2_ was added to each well at the indicated concentrations (0, 10000 and 30000 μM for HCC95; 0, 500 and 1000 μM for HCC15) and the cells were cultured for an additional 24 hours. Following the treatments, cells were washed with PBS and fixed with 4% formalin. The fixed cells were stained with 0.2% crystal violet. The rinsed and dried plates were scanned at a resolution of 600 dpi. Quantitative analysis was performed using Fiji (ImageJ distribution).^97^ Images were converted to 8-bit grayscale. Background correction was applied by dividing the experimental image by an empty plate image, and the signal intensity was adjusted using the “Multiply” function. The resulting images were inverted so that higher pixel intensity corresponded to higher cell density. The Integrated Density (IntDen) was measured within circular ROIs of equal area for each well. Cell viability was calculated as the percentage of IntDen relative to the control wells. Statistical significance was determined using Student’s t-test with Holm-Bonferroni correction.

#### Western blot analysis

Cells were seeded into 10-cm dishes at a density of 1 × 10^6^ cells/dish for both HCC95 and HCC15. After 24 hours of incubation, the culture medium was replaced with 0.5% FBS medium containing either 0.1% DMSO, 10 μM ICG001, or 150 nM PP. After 14 hours of pre-treatment, H_2_O_2_ was added to each dish at the indicated concentrations (0 or 30000 μM for HCC95; 0 or 1000 μM for HCC15), and the cells were cultured for an additional 10 hours. Following the treatments, cells were washed with cold PBS, and total cell lysates were extracted using a lysis buffer containing RIPA buffer, phosphatase inhibitor cocktails 2 and 3 (Sigma-Aldrich), and cOmplete Mini Protease Inhibitor Cocktail (Roche). Equal amounts of protein were separated by SDS-PAGE and transferred onto membranes. The membranes were incubated overnight at 4°C with the following primary antibodies: WNT3, β-catenin, c-Myc, Cyclin D1, Cleaved PARP, and GAPDH (loading control). The membranes were then washed with TBS and incubated with Anti-rabbit IgG, HRP-linked Antibody (Cell Signaling Technology) for 60 minutes at room temperature. Specific signals were detected using the Amersham ECL Prime Western Blotting Detection Reagent (Cytiva) and visualized using a LuminoGraph II (ATTO). Full uncropped images are provided in Figure S6A.

### Quantification and statistical analysis

Differential expression analysis of scRNA-seq data was performed using the Wilcoxon rank-sum test with BH correction, as implemented in Scanpy’s sc.tl.rank_genes_groups() function. DEGs were defined by log2FC > 2 and FDR <0.05. ORA was performed using GSEApy’s enrichr() function, which applies Fisher’s exact test with BH correction by default; terms with FDR <0.05 were considered significant. Ligand–receptor interactions were inferred using CellPhoneDB’s default permutation-based statistical framework (P < 0.05). For spatial transcriptomics, comparisons of cell-type abundance and gene expression levels between within-UIP and outside-UIP regions were conducted using the Wilcoxon rank-sum test; P < 0.05 was considered statistically significant. Connectivity analysis thresholds were defined as described in the signature connectivity mapping section above. For *in vitro* cell viability assays, comparisons between groups were performed using Student’s t-test followed by the Holm-Bonferroni correction, and an adjusted P < 0.05 was considered statistically significant.

## Supplemental information

Document S1. Figures S1–S6 and Table S1

Table S2. LUSC3-up and -down signatures used for connectivity analysis
