# Supplementary material for: Wnt3-mediated fibrosis and carcinogenesis of lung squamous cell carcinoma in idiopathic pulmonary fibrosis
Source: iScience. 2026 Apr 9;29(5):115667. doi: 10.1016/j.isci.2026.115667 (PMC13127482; doi:10.1016/j.isci.2026.115667)
Supplement: Document S1. Figures S1–S6 and Table S1 [file mmc1.pdf]

iScience, Volume *29*

Supplemental information

Wnt3-mediated fibrosis and carcinogenesis of lung squamous cell carcinoma in idiopathic pulmonary fibrosis

Atsushi Matsuoka, Kazuhiko Shien, Shuta Tomida, Masayoshi Ohki, Hidejiro Torigoe, Kazuya Hisamatsu, Ryota Fujiwara, Kosei Ishimura, Shunsuke Mori, Ryunosuke Fujii, Asuka Mimata, Kazuhiro Okada, Ryo Yoshichika, Mao Yoshikawa, Yuma Fukumoto, Haruchika Yamamoto, Kumi Nakajima, Shin Tanaka, Ken Suzawa, Kentaroh Miyoshi, Mikio Okazaki, Seiichiro Sugimoto, Hirofumi Inoue, Daisuke Ennishi, and Shinichi Toyooka

Table S1: Patient characteristics and status of experiment

Pt.	Tumor location	Experiment	Age	Gender	Pack years	VC (ml)	%VC	Histology	pStage
35	Within UIP	Spa	56	Male	82.5	4530	122.4	LUSC	IA2
36	Within UIP	Scr, WES	64	Male	47	4210	128.7	LUSC	IIB
38	Within UIP	Spa, WES	56	Male	36	4130	109.8	LUSC	IA2
47	Within UIP	Spa, WES	66	Male	58.5	2830	75.7	LUSC	IB
44	Outside UIP	Scr, Spa, WES	79	Male	43	2020	70.1	LUSC	IB
50	Outside UIP	Spa, WES	74	Male	70	2920	76.2	LUSC	IIIB
51	Outside UIP	Spa, WES	74	Male	54	3490	102.9	LUSC	IB

Spa: Spatial transcriptomics, Scr: Single-cell RNA sequencing, WES: Whole-exome sequencing

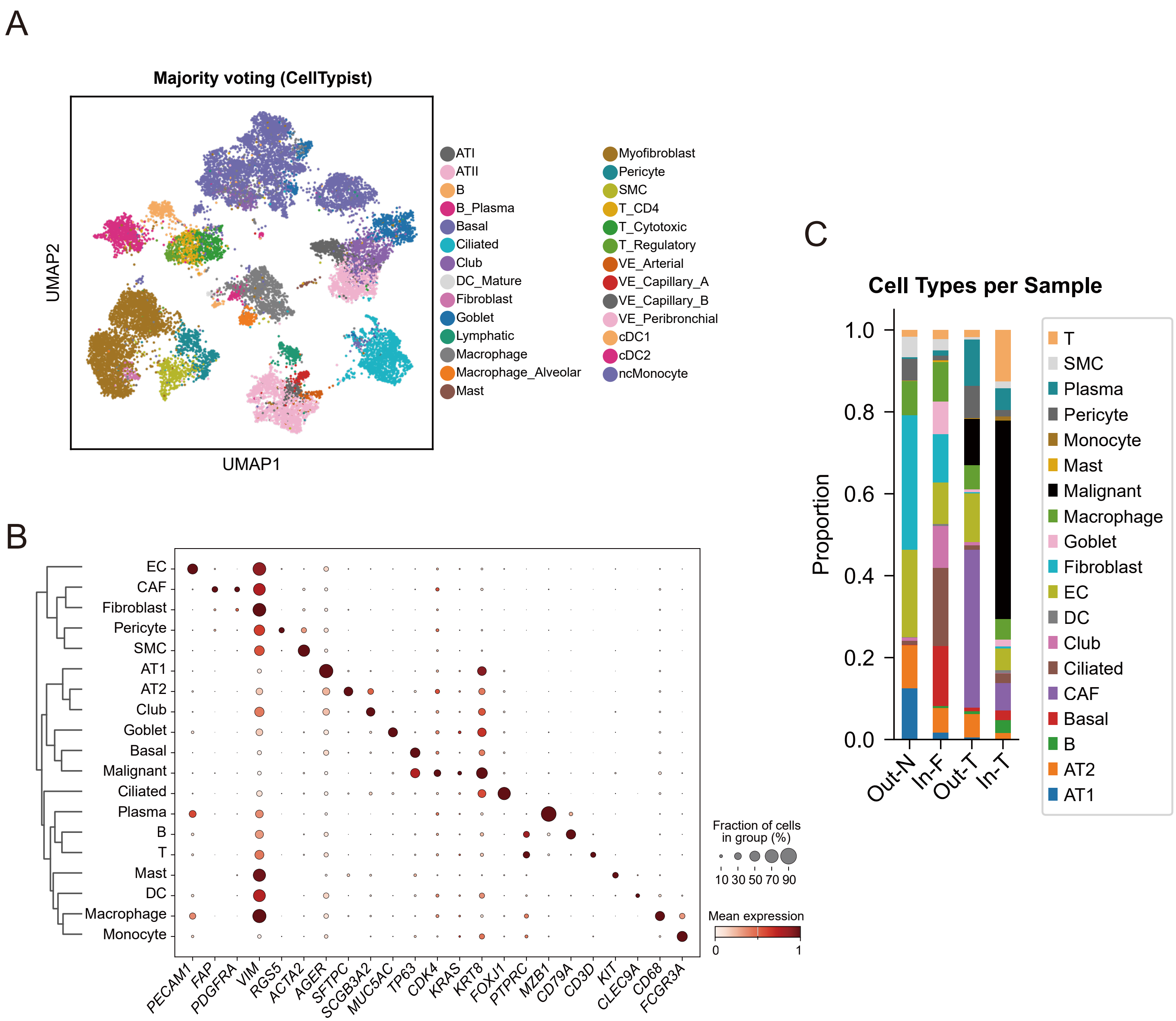

Figure S1

A) UMAP plot showing preliminary cell type annotations performed using CellTypist with the *Human_IPF_Lung.pkl* model, which includes reference cell types derived from idiopathic pulmonary fibrosis (IPF), chronic obstructive pulmonary disease (COPD), and healthy adult lung tissues. Cell labels were assigned using the celltypist.annotate() function with the majority_voting argument, which assigns a consensus label to each Leiden cluster based on the most frequent prediction among its constituent cells. As the reference model does not include tumor-derived cells, malignant cells were not annotated.

B) Dot plot displaying the expression of established marker genes across annotated cell types to support and refine manual cell type assignments. Dot size represents the proportion of cells expressing each gene within a given cell type, and color intensity indicates the scaled expression level among those expressing cells.

C) Bar plot depicting the relative abundance of each annotated cell type across the four scRNA-seq samples.

A

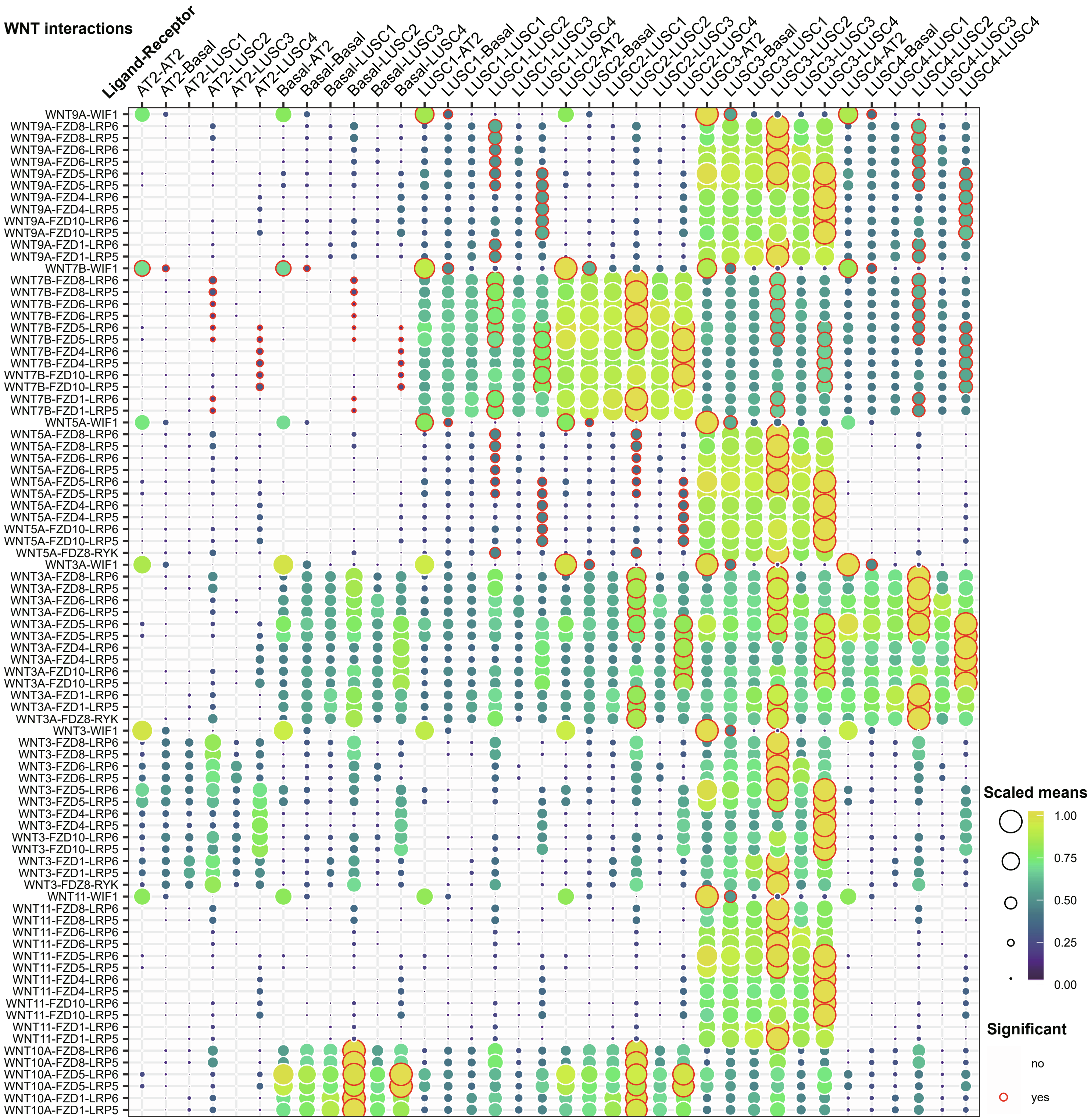

Figure S2

A) Wnt-related ligand–receptor interactions. Shown are Wnt ligand–receptor pairs that exhibited statistically significant interactions ($P < 0.05$ in at least one pairwise comparison) among epithelial populations, including LUSC1–LUSC4 (tumor subclones), Basal, and AT2 cells (used as lung tissue controls). Dot size and color represent the scaled mean expression of each interaction, while red outlines indicate statistical significance.

A

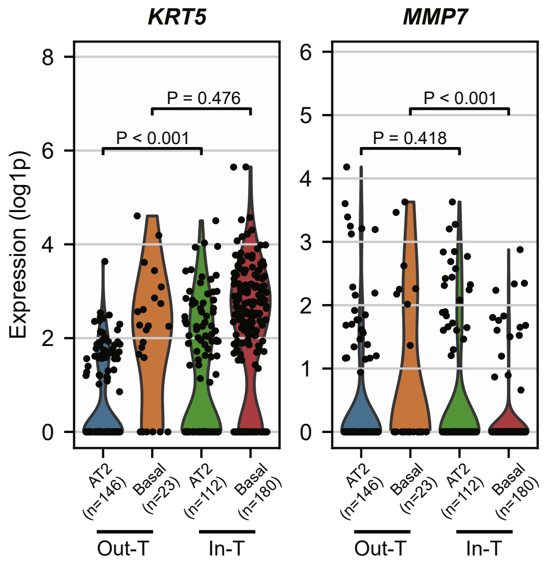

Figure S3

A) Violin plots showing the expression of *KRT5* and *MMP7* in Basal and AT2 cells from tumor tissues. Statistical significance was determined by Wilcoxon rank-sum test; exact P values and the number of cells (n) are indicated.

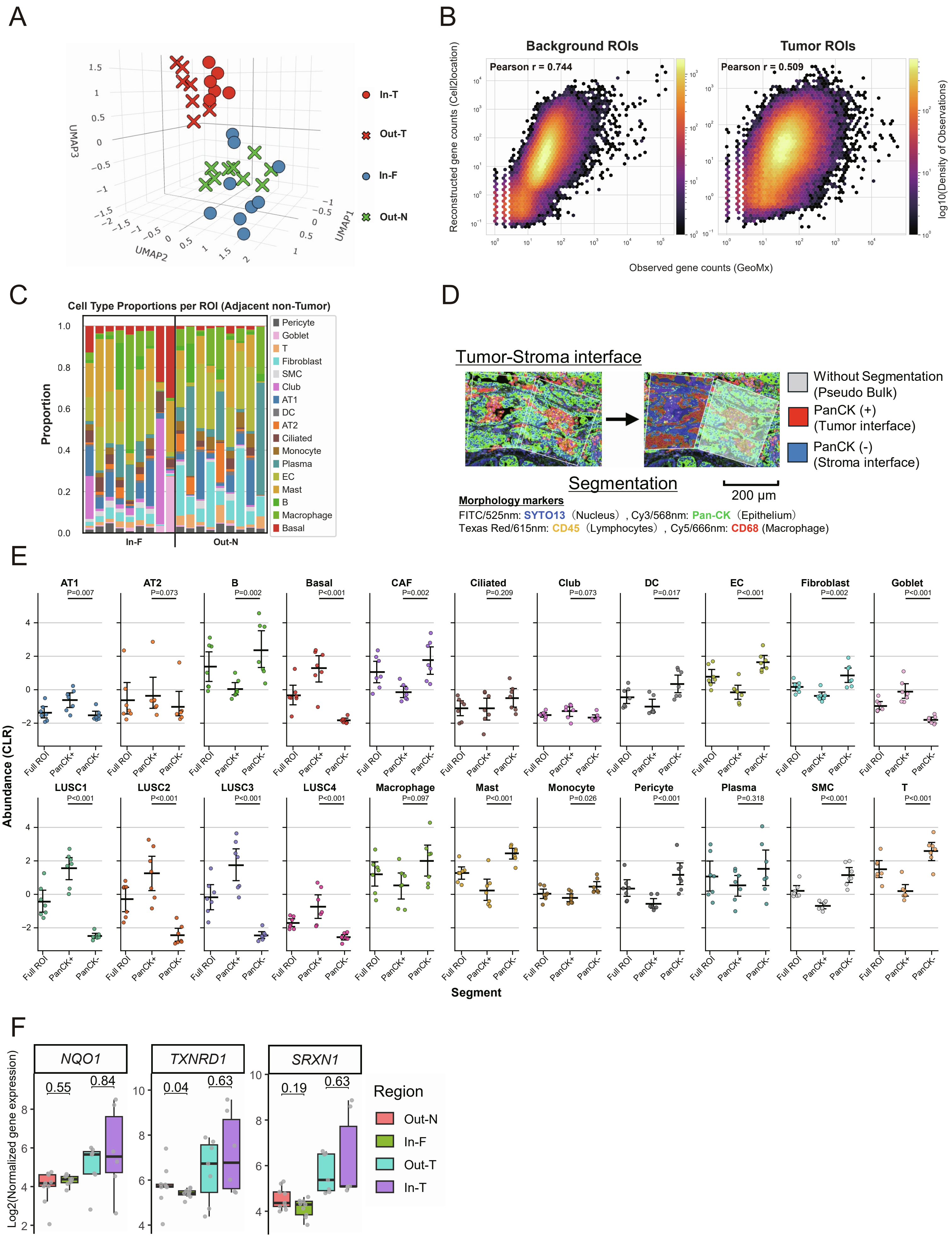
A
In-T
Out-T
In-F
Out-N
UMAP1
UMAP2
UMAP3
B
Background ROIs
Tumor ROIs
Pearson r = 0.744
Pearson r = 0.509
Reconstructed gene counts (Cell2location)
Observed gene counts (GeoMx)
log10(Density of Observations)
C
Cell Type Proportions per ROI (Adjacent non-Tumor)
Proportion
In-F
Out-N
Pericyte
Goblet
T
Fibroblast
SMC
Club
AT1
DC
AT2
Ciliated
Monocyte
Plasma
EC
Mast
B
Macrophage
Basal
D
Tumor-Stroma interface
Segmentation
Without Segmentation (Pseudo Bulk)
PanCK (+) (Tumor interface)
PanCK (-) (Stroma interface)
200 μm
Morphology markers
FITC/525nm: SYTO13（Nucleus）, Cy3/568nm: Pan-CK（Epithelium）
Texas Red/615nm: CD45（Lymphocytes）, Cy5/666nm: CD68 (Macrophage)
E
AT1 P=0.007
AT2 P=0.073
B P=0.002
Basal P<0.001
CAF P=0.002
Ciliated P=0.209
Club P=0.073
DC P=0.017
EC P<0.001
Fibroblast P=0.002
Goblet P<0.001
LUSC1 P<0.001
LUSC2 P<0.001
LUSC3 P<0.001
LUSC4 P<0.001
Macrophage P=0.097
Mast P<0.001
Monocyte P=0.026
Pericyte P<0.001
Plasma P=0.318
SMC P<0.001
T P<0.001
Full ROI
PanCK+
PanCK-
Abundance (CLR)
Segment
F
NQO1
TXNRD1
SRXN1
0.55
0.84
0.04
0.63
0.19
0.63
Log2(Normalized gene expression)
Region
Out-N
In-F
Out-T
In-T

Figure S4

A) 3D UMAP of tumor core and adjacent non-tumor ROIs based on global gene expression. UMAP was performed using all available genes.

B) Assessment of model reconstruction accuracy. Scatter plots comparing observed gene counts in GeoMx ROIs (x-axis) with reconstructed counts inferred by Cell2location (y-axis). Reconstructed counts were calculated as the product of estimated cell abundance, scRNA-seq–derived reference signatures, and ROI-specific detection efficiency. Pearson correlation coefficients (r) are shown.

C) Stacked 100% bar plot depicting the relative cell-type composition of each reference cell type in the In-F or Out-N ROIs. Bars represent individual ROIs and are grouped accordingly. Cell-type abundances were inferred using the Cell2location-WTA model with a non-malignant cell type reference (LUSC1–4 and CAF were excluded). Segment height indicates the proportion of each cell type.

D) Representative immunofluorescent images showing the segmentation strategy used for tumor ROIs in spatial transcriptomic analysis. Tumor regions were segmented based on Pan-CK expression to define epithelial (Pan-CK^{+}; tumor interface, red overlay) and stromal (Pan-CK^{-}; stroma interface, blue overlay) compartments within each ROI. This compartment-specific segmentation was performed using GeoMx DSP to distinguish transcriptomic profiles of the tumor and tumor microenvironment. Additionally, adjacent regions were selected as pseudo-bulk ROIs without segmentation (white overlay).

E) Cell-type abundance across tumor–stroma interface ROIs. Each panel shows one of the 22 reference cell types. Plots display centered log-ratio (CLR)–transformed expected counts in three segment classes: Full ROI (pseudo-bulk), Pan-CK^{+} (tumor interface), and Pan-CK^{-} (stroma interface). Horizontal bars represent the mean, and error bars indicate the 95% bootstrapped confidence interval (CI) calculated from 1000 iterations. Individual dots represent each ROI. For each cell type, the P-value from a Wilcoxon rank-sum test comparing Pan-CK^{+} and Pan-CK^{-} segments is shown above the bracket. As expected, tumor subclones (LUSC1–4) are significantly enriched in Pan-CK^{+} regions, whereas most immune and mesenchymal populations are significantly higher in Pan-CK^{-} regions. In most lineages, the mean of the Full ROI lies between the two compartment-specific means, reflecting the mixture of epithelial and stromal transcriptomes. These results confirm that our deconvolution workflow performs accurately.

F) Expression of LUSC3-specific KEAP1–NFE2L2 pathway targets across spatial regions. Boxplots show normalized gene expression levels of *NQO1*, *TXNRD1*, and *SRXN1* across four classes. Individual ROIs are plotted as gray dots with jitter. Pairwise comparisons (Out-N vs In-F; Out-T vs In-T) were evaluated using the Wilcoxon rank-sum test, and *P*-values are indicated above each comparison. Although the In-T group tended to have higher average expression levels, the high variability within the region and the limited number of GeoMx ROIs (“local-bulk” profiling) did not yield statistically significant differences in pathway upregulation, which appears to depend on the presence of the LUSC3 subclone.

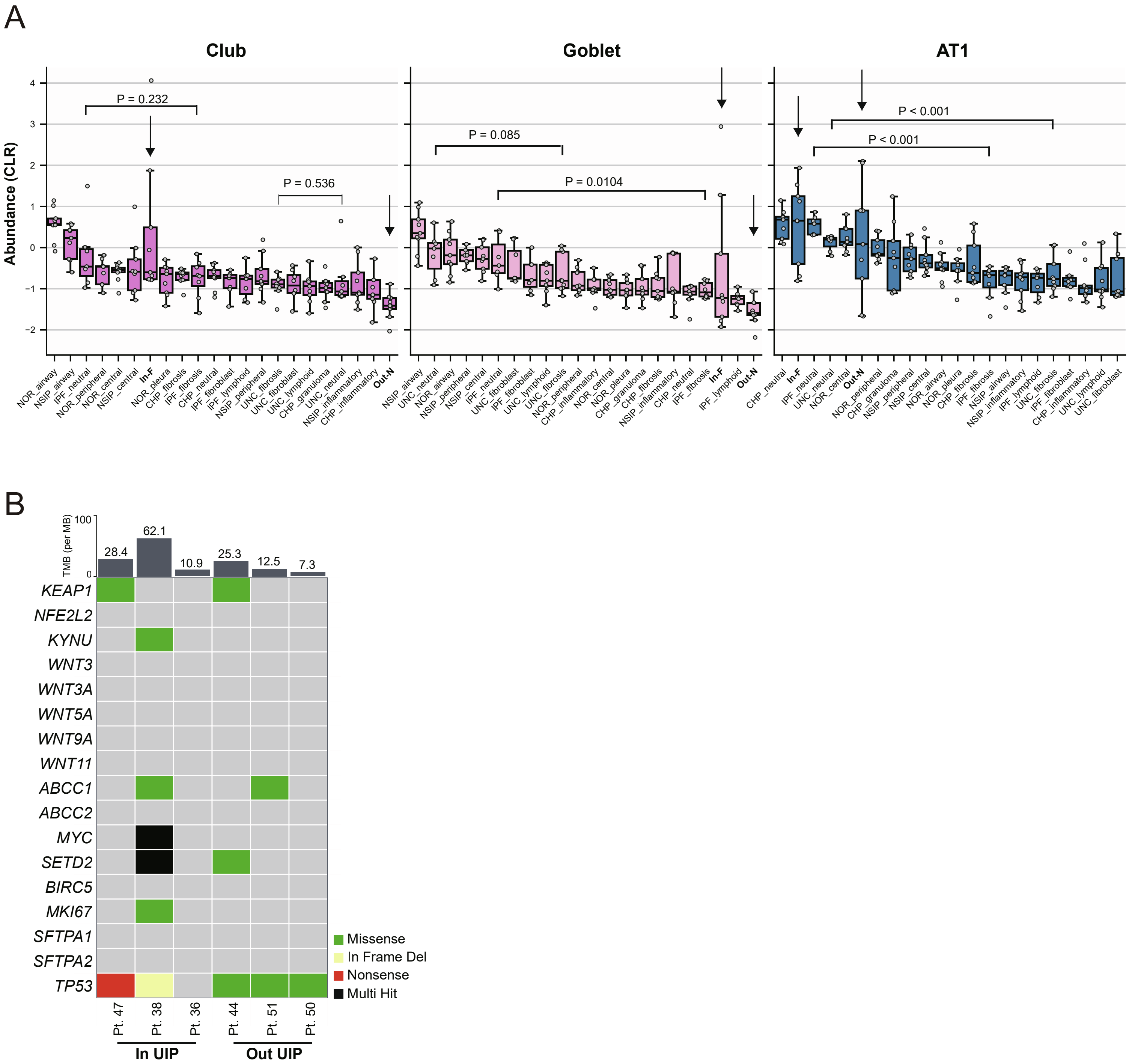
A
Club
Goblet
AT1
Abundance (CLR)
P = 0.232
P = 0.536
P = 0.085
P = 0.0104
P < 0.001
P < 0.001
In-F
Out-N
NOR_airway
NSIP_airway
IPF_neutral
NOR_peripheral
NOR_central
NSIP_central
NOR_pleura
CHP_fibrosis
IPF_fibrosis
CHP_neutral
IPF_fibroblast
IPF_lymphoid
NSIP_peripheral
UNC_fibrosis
UNC_fibroblast
UNC_lymphoid
CHP_granuloma
UNC_neutral
NSIP_inflammatory
CHP_inflammatory
B
TMB (per MB)
28.4
62.1
10.9
25.3
12.5
7.3
KEAP1
NFE2L2
KYNU
WNT3
WNT3A
WNT5A
WNT9A
WNT11
ABCC1
ABCC2
MYC
SETD2
BIRC5
MKI67
SFTPA1
SFTPA2
TP53
Missense
In Frame Del
Nonsense
Multi Hit
Pt. 47
Pt. 38
Pt. 36
Pt. 44
Pt. 51
Pt. 50
In UIP
Out UIP

Figure S5

A) Club cell, Goblet cell, and AT1 cell abundance across lung microenvironments. Boxplots display CLR-transformed expected counts for histological microenvironments present in our LUSC-UIP dataset (adjacent non-tumor ROIs) and the external ILD dataset GSE255174. Categories are ordered by the median value within each panel. Statistical test (Wilcoxon rank-sum test) results are shown between the fibrotic and uninvolved regions in IPF and UNC. In-F and Out-N classes were derived from our study. Each individual gray dot represents a single ROI; boxes denote the median and interquartile range, and whiskers extend to 1.5 × IQR.

IPF: idiopathic pulmonary fibrosis; NSIP: non-specific interstitial pneumonia; CHP: chronic hypersensitivity pneumonitis; UNC: unclassifiable fibrosing interstitial lung diseases; NOR: normal control; fibrosis: fibrosis area; fibroblast: fibroblastic foci; lymphoid: lymphoid aggregate area; neutral: fibrosis-uninvolved area; peripheral: peripheral lung fibrosis; central: central lung fibrosis; inflammatory: inflammatory area; airway: bronchial area; granuloma: granuloma area; pleura: pleural region.

B) Mutation profiles of key genes highlighted in the Discussion section. Bar plots at the top represent the tumor mutation burden (TMB) for each patient. The heatmap below shows the mutation status of selected genes. Mutation types are color-coded, and gray indicates no detected mutation.

A

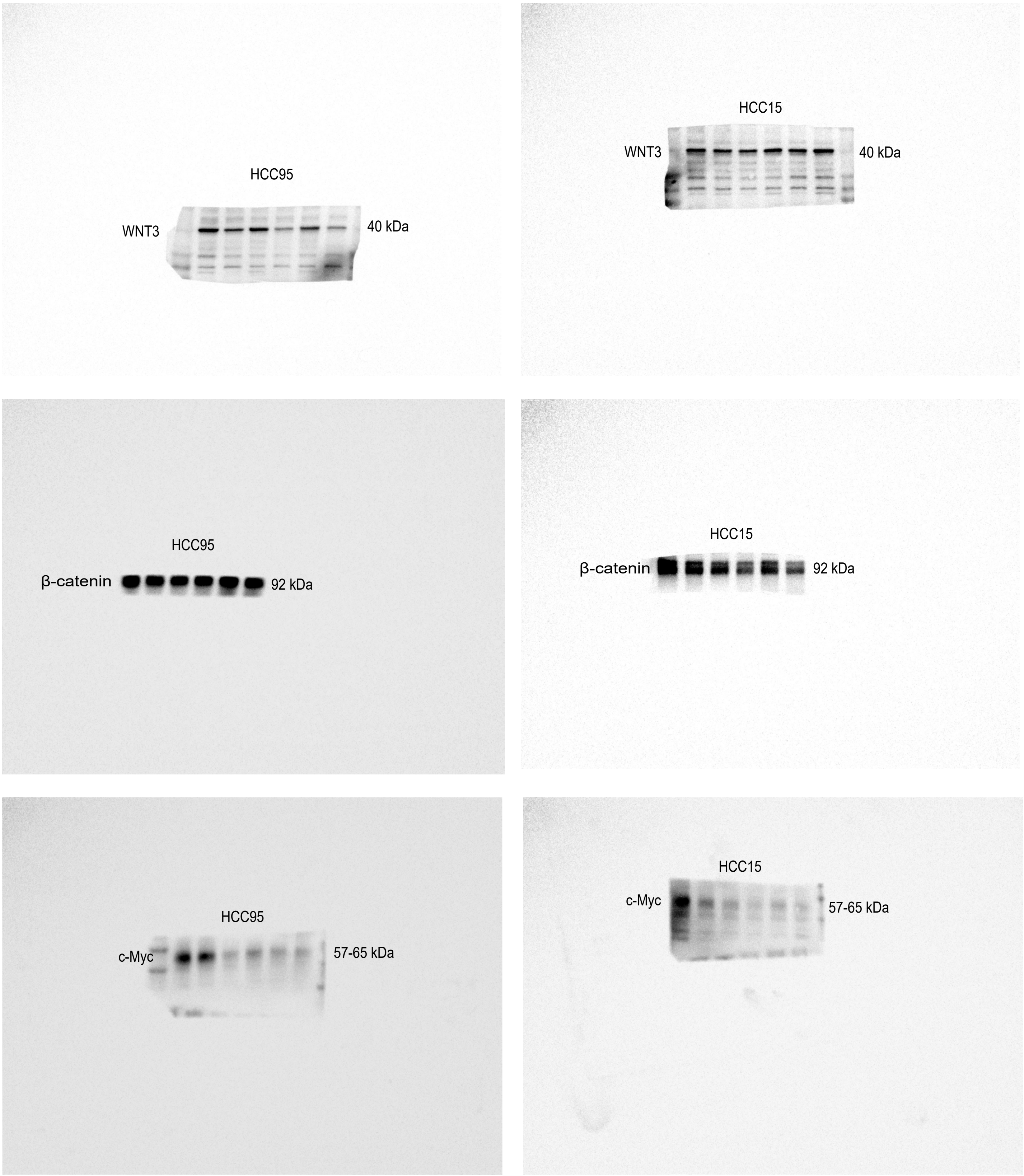
HCC95
WNT3
40 kDa
HCC15
WNT3
40 kDa
HCC95
β-catenin
92 kDa
HCC15
β-catenin
92 kDa
HCC95
c-Myc
57-65 kDa
HCC15
c-Myc
57-65 kDa

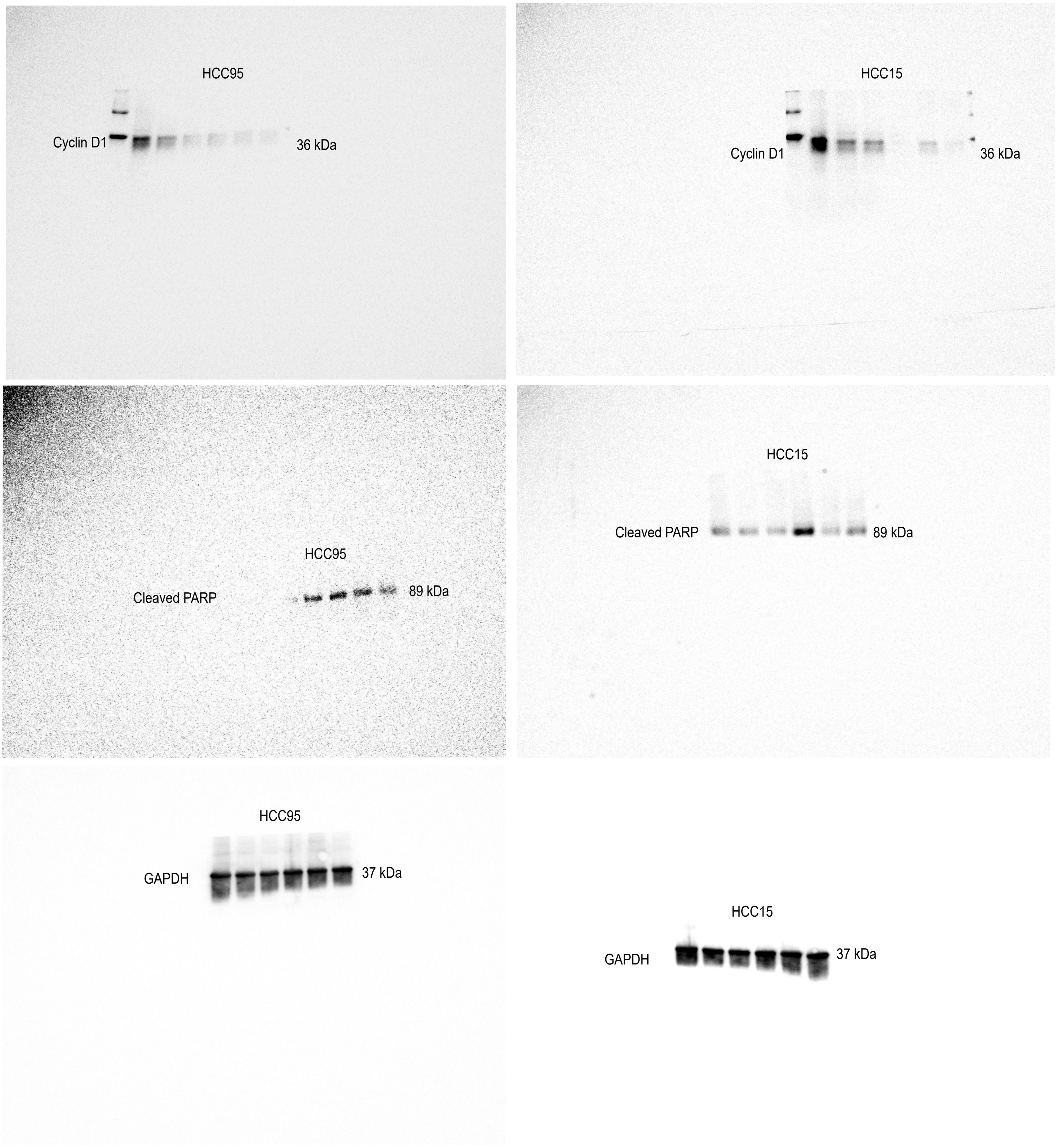

Figure S6

A) Uncropped and unedited Western blot images.
